# ATR inhibition radiosensitizes cells through augmented DNA damage and G_2_ cell cycle arrest abrogation

**DOI:** 10.1172/jci.insight.179599

**Published:** 2024-10-08

**Authors:** Scott J. Bright, Mandira Manandhar, David B. Flint, Rishab Kolachina, Mariam Ben Kacem, David K.J. Martinus, Broderick X. Turner, Ilsa Qureshi, Conor H. McFadden, Poliana C. Marinello, Simona F. Shaitelman, Gabriel O. Sawakuchi

**Affiliations:** 1 Department of Radiation Physics, The University of Texas MD Anderson Cancer Center, Houston, Texas, USA.; 2 Department of Biosciences, Rice University, Houston, Texas, USA.; 3 Department of Immunology, The University of Texas MD Anderson Cancer Center, Houston, Texas, USA.; 4 Emory University School of Medicine, Emory University, Atlanta, Georgia, USA.; 5 Department of Breast Radiation Oncology, The University of Texas MD Anderson Cancer Center, Houston, Texas, USA.

**Keywords:** Oncology, Cell cycle, DNA repair, Radiation therapy

## Abstract

Ataxia telangiectasia and Rad3-related protein (ATR) is a key DNA damage response protein that facilitates DNA damage repair and regulates cell cycle progression. As such, ATR is an important component of the cellular response to radiation, particularly in cancer cells, which show altered DNA damage response and aberrant cell cycle checkpoints. Therefore, ATR’s pharmacological inhibition could be an effective radiosensitization strategy to improve radiotherapy. We assessed the ability of an ATR inhibitor, AZD6738, to sensitize cancer cell lines of various histologic types to photon and proton radiotherapy. We found that radiosensitization took place through persistent DNA damage and abrogated G_2_ cell cycle arrest. We also found that AZD6738 increased the number of micronuclei after exposure to radiotherapy. We found that combining radiation with AZD6738 led to tumor growth delay and prolonged survival relative to radiation alone in a breast cancer model. Combining AZD6738 with photons or protons also led to increased macrophage infiltration at the tumor microenvironment. These results provide a rationale for further investigation of ATR inhibition in combination with radiotherapy and with other agents such as immune checkpoint blockade.

## Abstract 2

Inhibition of ATR sensitizes cancer cells to photons and protons. The combination improved survival and augmented antitumor immune signaling in a breast cancer model.

## Graphical abstract

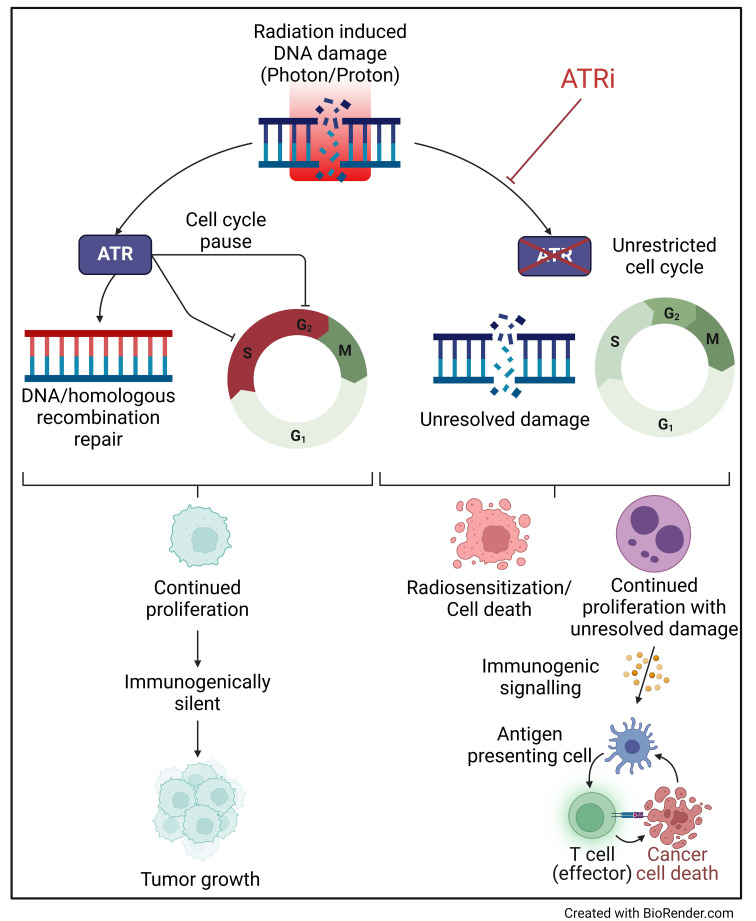

## Introduction

Radiotherapy is a key treatment modality in cancer therapy. However, many tumors are inherently resistant or develop resistance over time. Therefore, there is an interest in developing strategies that improve the effectiveness of radiotherapy. One such approach is to combine radiotherapy with radiosensitizers, including small molecule inhibitors that inhibit DNA damage response and DNA repair. The protein kinase ataxia telangiectasia and Rad3-related (ATR) is an attractive target because it has roles in DNA damage response and cell cycle checkpoint regulation (1, 2). Cells depend heavily on ATR to process DNA lesions that are encountered in replicating DNA, such as single-ended DNA double–strand break lesions and stalled replication forks, in addition to pausing cell cycle at the G_2_ phase (3, 4). These responses are important for cell survival following radiation exposure. Therefore, targeting ATR with pharmacological inhibitors is an attractive approach to radiosensitize cancer cells (5–13).

Inhibiting ATR is of particular interest because of its central role in activating G_2_ cycle arrest, which cancer cells rely on heavily, as the G_1_ checkpoint is often defective (1, 14). Therefore, inhibiting ATR is expected to radiosensitize more cancer cells, which have defective G_1_ checkpoint, than normal cells, which have intact G_1_ checkpoint. Furthermore, ATR’s role in DNA repair, specifically promoting homologous recombination (HR), may be leveraged with different radiation modalities such as protons, carbon ions, and α particles, which are known to increase the reliance on the HR pathway (15–22).

These particle beams have a greater relative biological effectiveness (RBE), which has been attributed to their higher ionization density or linear energy transfer (LET). The greater ionization density leads to clustering of ionizing events, which, in DNA, can induce multiple lesions in close proximity. A leading hypothesis is that damage clustering leads to difficulty in assembling repair machinery, consequently causing more cell death (15, 23, 24).

The combined effects of increased DNA damage and dysregulated cell cycle progression in irradiated cells also increases the formation of micronuclei (MN) (5), which are known to activate the cyclic GMP–AMP synthase/stimulator of interferon genes (cGAS/STING) pathway and in turn activate antitumor immune responses (8, 25–29).

Thus, the combination of ATR inhibition and radiotherapy, specifically proton radiotherapy, is an attractive one for tumor-specific radiosensitization. There are currently numerous ATR inhibitors in clinical trials as monotherapy or combined with other systemic therapies (ClinicalTrials.gov NCT05798611, NCT05269316, NCT04514497, and NCT02264678) as well as combined with radiation (NCT04576091, NCT05566574). Here, we investigated the mechanistic basis for ATR-induced radiosensitization in the context of photon and proton exposures, which is currently underexplored, and whether combinations could augment antitumor immunity. We showed that the ATR inhibitor AZD6738 effectively radiosensitizes cells across multiple histologic types. We corroborated that ATR inhibition could have profound effects on cell cycle and DNA damage response and repair and that these were likely the cause of radiosensitization to both photons and protons. We showed that this combination was effective in vivo using an aggressive breast cancer model, which also showed differences in immune infiltration.

## Results

### AZD6738 radiosensitizes both normal and cancer cells to photons and protons.

**Figure 1:**
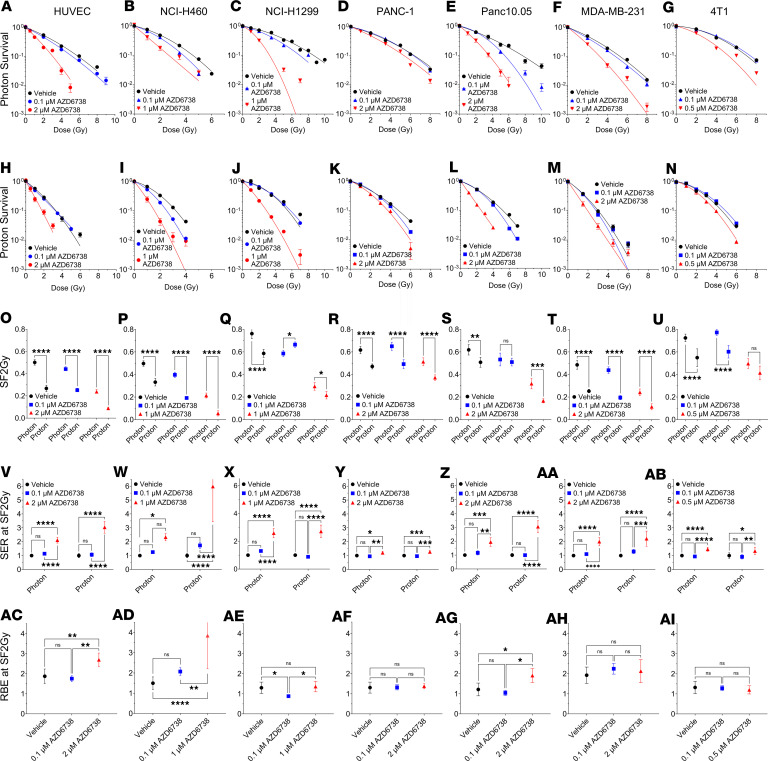
AZD6738 is an effective radiosensitizer in vitro. Survival in human umbilical vein endothelial cells (HUVEC) (**A** and **H**), NCI-H460 (**B** and **I**), NCI-H1299 (**C** and **J**), PANC-1 (**D** and **K**), Panc10.05 (**E** and **L**), MDA-MB-231 (**F** and **M**), and 4T1 (**G** and **N**) following photon and proton exposure. Surviving fractions of cells after 2 Gy (SF2Gy) of photons or protons. (**O**) HUVEC, (**P**) NCI-H460, and (**Q**) NCI-H1299 lung cancer cells, (**R**) PANC-1 and (**S**) Panc10.05 pancreatic cancer cells, and (**T**) MDA-MB-231 and (**U**) 4T1 breast cancer cells. (**V**–**AB**) For each radiation type, the sensitization enhancement ratio (SER) at SF2Gy was calculated relative to the control. (**AC**–**AI**) The relative biological effectiveness at SF2Gy (RBE at SF2Gy) of protons for each drug concentration was calculated relative to photons. A minimum of 3 repeats were performed (Supplemental Table 1); error bars represent the SD. Statistical significance was assessed with 2-way ANOVA with Tukey’s multiple-comparison test (**A**–**AB**) or 1-way ANOVA with Tukey’s multiple-comparison test (**AC**–**AI**). NS, non-significant. *P < 0.05, **P < 0.01, ***P < 0.001, ****P < 0.0001. Parts of Figure 1 were copied from Bright et al. (7) with permission from Radiation Research (© 2023 Radiation Research Society).

We first examined the ability of AZD6738 to radiosensitize several human cancer cell lines from different cell types, including lung cancer (NCI-H460 and NCI-H1299), pancreatic cancer (PANC-1 and PANC10.05), and breast cancer (MDA-MB-231), and a normal cell line (human umbilical vein endothelial cell, HUVEC); we also tested its effects on a mouse model of triple-negative breast cancer, 4T1 (Figure 1, A–N). We chose a broad range of cell lines to ensure that our results were not biased regarding anatomic site and to characterize heterogeneity in response across cell lines. AZD6738 was used at various concentrations, including a low dose (0.1 μM) that had minimal effects on plating efficiency, and a higher dose that depended on the cell line (0.5–2 μM) (Supplemental Figure 1; supplemental material available online with this article; https://doi.org/10.1172/JCI179599DS1). As expected, protons demonstrated a greater RBE, with a lower surviving fraction at 2 Gy (SF2Gy) relative to photons (Figure 1, O–U). Note that a high LET, which is greater than that achieved clinically within a tumor, was used for most of the in vitro proton irradiations. However, we also showed an RBE (at D_10%_) greater than unity (1.11 ± 0.02, mean ± SD) for the 4T1 cell line for a LET of 3.85 keV/μm, which is typically the LET within the tumor (Supplemental Figure 2). When cells were treated with AZD6738 at concentrations greater than 0.5 μM, all cells showed increased radiosensitization (Figure 1, V–AB), with the greatest effect observed in NCI-H460 (Figure 1W) treated with 1 μM AZD6738 (a 6.0 ± 2.5-fold increase in sensitivity to protons, mean ± SD). HUVEC was significantly radiosensitized to both photons and protons (Figure 1V). The RBE was calculated for vehicle and AZD6738 groups using SF2Gy (which was fixed across cell lines) and found that HUVEC, NCI-H460, and Panc10.05 showed significant increases in RBE at SF2Gy (Figure 1, AC, AD, and AG). Increased RBE at SF2Gy for cells treated with AZD6738 was also observed for a clinically relevant LET (3.85 keV/μm) for the 4T1 cell line (Supplemental Figure 2). No other cell lines showed increased RBE at SF2Gy when protons were combined with AZD6738 (Figure 1, AE, AF, AH, and AI). We also calculated RBE at D_50%_ and D_10%_, and similar trends were observed (Supplemental Tables 1–3). Similar cell survival trends were observed in NCI-H460 and NCI-H1299 following treatment with a different ATR inhibitor (BAY1895344) (Supplemental Figure 3), indicating that the ATR inhibitor effect is not AZD6738 specific.

### AZD6738 modulates cell cycling in response to photons and protons and increases residual DNA damage signaling.

**Figure 2:**
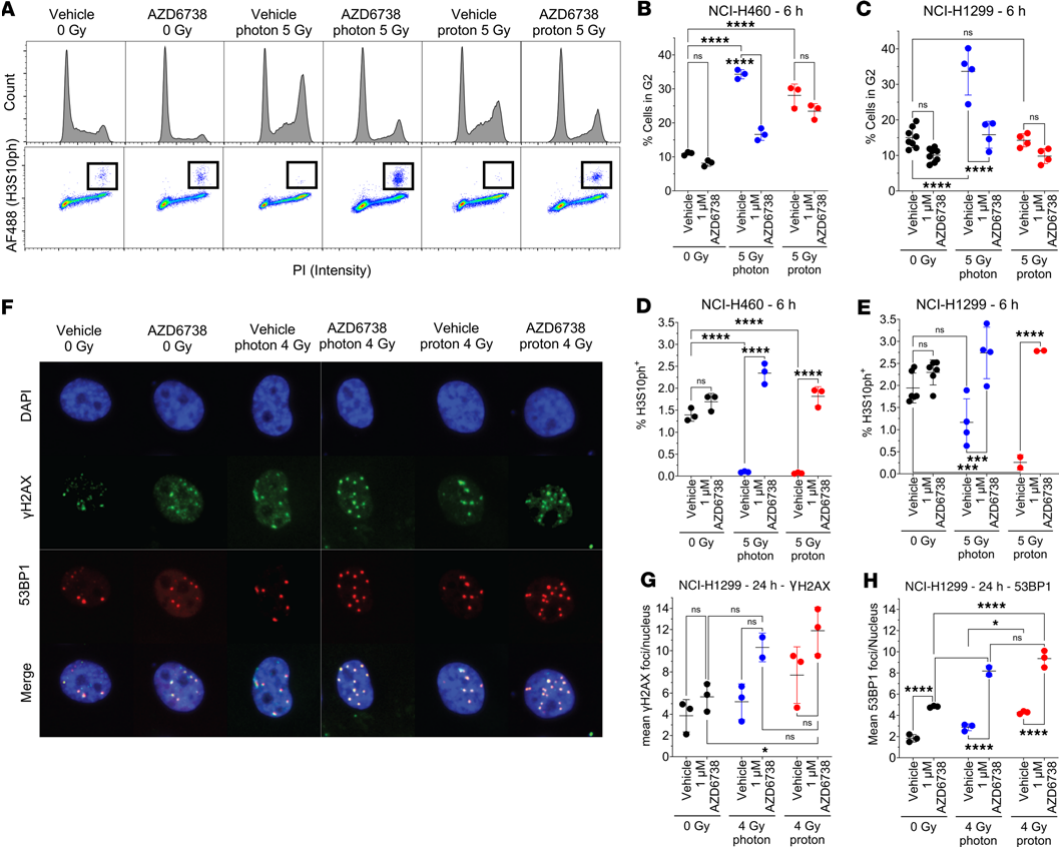
AZD6738 promotes G_2_-M transition after irradiation and increases residual DNA damage. (**A**) Top: Representative cell cycle distributions in NCI-H460 pretreated with AZD6738 for 6 hours before irradiation and analyzed 6 hours after radiation. Bottom: Representative mitotic populations determined by staining for histone H3 phosphorylated at serine 10 (H3S10ph). Inset squares illustrate H3S10ph-positive cells. (**B** and **C**) Percentages of cells in G_2_ were calculated with FlowJo v10.7 (BD Life Sciences) in (**B**) NCI-H460 cells and (**C**) NCI-H1299 cells. (**D** and **E**) Numbers of mitotic cells were calculated using FlowJo in (**D**) NCI-H460 cells and (**E**) NCI-H1299 cells. (**F**) Representative immunofluorescence images of foci in NCI-H1299 cells treated with AZD6738 1 hour before irradiation and analyzed for γH2AX and 53BP1 foci as a surrogate for DNA damage 24 hours after irradiation. Original magnification, ×20. (**G**) γH2AX and (**H**) 53BP1 foci in H1299 cells exposed to photons or protons. At least 10,000 cells were analyzed per group, and error bars represent the SD. Statistical significance was assessed with 2-way ANOVA with Tukey’s multiple-comparison test. NS, non-significant. *P < 0.05; ***P < 0.001; ****P < 0.0001.

Having observed enhanced cell killing upon combining AZD6738 with radiation, we next sought to determine how AZD6738 modulates the cell’s DNA damage response, both in terms of cell cycling and DNA damage repair. We observed that both photons and protons induced significant blockade at G_2_ in NCI-H460 and NCI-H1299 lung cancer cells (Figure 2, A–C). In the presence of AZD6738, NCI-H1299 cells showed significant reduction in the percentage of cells in G_2_, suggesting that ATR inhibition may overcome radiation-induced activation of this cell cycle checkpoint. Interestingly, AZD6738 overcame the blockade induced by photons, but not by protons. However, it efficiently increased the release of cells in mitosis when associated with photons and protons (relative to radiation plus vehicle), evaluated by using histone H3 phosphorylated at serine 10 (H3S10ph) labeling (Figure 2, D and E). In examining DNA damage signaling, we observed that radiation increased the number of γH2AX and 53BP1 foci present at 24 hours after exposure to 4 Gy and that AZD6738 significantly increased this effect. Protons seemed to lead to more persistent γH2AX foci (not significant) and led to more 53BP1 foci (significant) compared with photons (Figure 2, F–H).

### AZD6738 disrupts formation of RAD51 foci.

**Figure 3:**
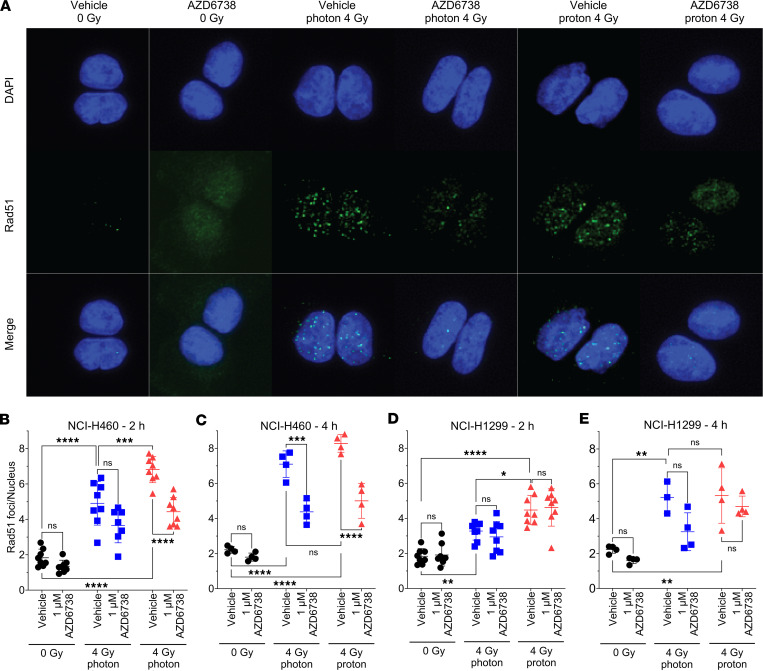
AZD6738 (1 μM) significantly reduces RAD51 foci formation in NCI-H460 cells, but not NCI-H1299 cells. (**A**) Representative images of RAD51 foci formation in NCI-H460 at 2 hours after irradiation. Original magnification, ×20. (**B** and **C**) RAD51 foci formation after photons and protons at (**B**) 2 hours and (**C**) 4 hours after irradiation of NCI-H460 cells. (**D** and **E**) RAD51 formation at (**D**) 2 hours or (**E**) 4 hours after irradiation with photons or protons. At least 3 independent repeats were performed. Error bars represent the SD. Statistical significance was assessed with 2-way ANOVA with Tukey’s multiple-comparison test. NS, non-significant. *P < 0.05; **P < 0.01; ***P < 0.001; ****P < 0.0001.

To clarify the effects of ATR inhibition specifically on DNA repair pathways, we tested the effects of AZD6738 on HR by investigating RAD51 formation in NCI-H460 and NCI-H1299 lung cancer cells (Figure 3A). Because the kinetics of HR repair and RAD51 foci formation are delayed relative to nonhomologous end-joining (NHEJ) repair, we tested RAD51 foci formation at 2 and 4 hours after irradiation. Experiments were performed with a higher LET (9.9 keV/μm) than what is typically found within tumors to allow us to maximize the physical differences to 6 MV x-rays. We observed that in both NCI-H460 and NCI-H1299 cells, treatment with protons alone led to more RAD51 foci than treatment with photons alone at 2 hours (Figure 3, B and D). This pattern was similar, although not statistically significant, at 4 hours after irradiation (Figure 3, C and E). NCI-H460 cells treated with 1 μM AZD6738 showed significant reductions in numbers of RAD51 foci in response to protons at 2 and 4 hours, but reductions in RAD51 foci after photons were evident only at 4 hours (Figure 3, B and C). Interestingly, AZD6738 had no significant effects on RAD51 foci formation in NCI-H1299 lung cancer cells, although the RAD51 foci number did decrease at 4 hours after irradiation. Similar RAD51 foci trends were observed in NCI-H460 and NCI-H1299 following treatment with a different ATR inhibitor (BAY1895344) (Supplemental Figure 3), supporting the notion that the ATR inhibitor effect is not AZD6738 specific.

### AZD6738 slows down γH2AX and 53BP1 foci kinetics after irradiation.

**Figure 4:**
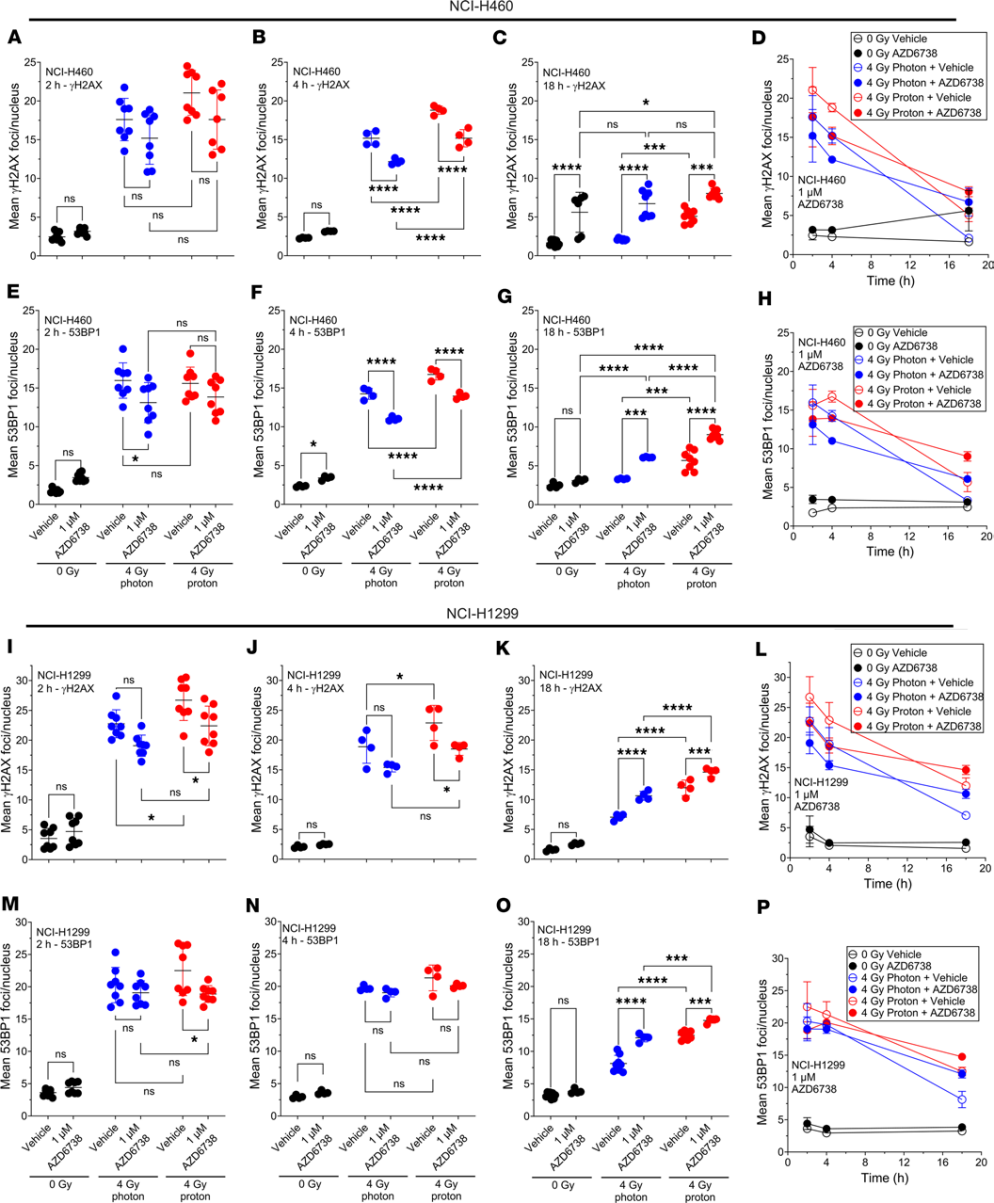
AZD6738 delays γH2AX and 53BP1 foci kinetics after irradiation, with protons having the greatest delay. γH2AX and 53BP1 foci were quantified as a function of time in NCI-H460 (**A**–**H**) and NCI-H1299 (**I**–**P**) at 2, 4, and 18 hours after irradiation with 4 Gy of 6 MV x-rays (photons) or 9.9 keV/μm protons. At least 3 independent repeats were performed. Error bars represent the SD. Statistical significance was assessed with 2-way ANOVA with Tukey’s multiple-comparison test. NS, non-significant. *P < 0.05; ***P < 0.001; ****P < 0.0001.

We also examined γH2AX and 53BP1 foci kinetics in these cell lines as a surrogate to assess impaired double-strand break (DSB) DNA repair (Figure 4). We observed a trend for fewer γH2AX foci in cells treated with radiation (photons or protons) and AZD6738 compared with radiation alone at 2 hours after irradiation, with significantly fewer γH2AX foci for the NCI-H1299 cell line treated with protons and AZD6738 compared with protons alone (Figure 4, A and I). At 4 hours after irradiation, we observed significantly fewer γH2AX foci in cells treated with radiation (photons or protons) and AZD6738 compared with radiation alone (Figure 4, B and J), which was more evident in NCI-H460 cells. At 18 hours after irradiation, we observed significantly more γH2AX foci in cells treated with radiation (photons or protons) and AZD6738 compared with radiation alone (Figure 4, C and K). We found that γH2AX foci number decreased as a function of time, with the slowest relative decrease observed for protons combined with AZD6738 (Figure 4, D and L). γH2AX foci resolved more slowly when cells were treated with radiation and AZD6738 compared with radiation alone.

We observed reduced 53BP1 foci numbers in cells treated with radiation (photons or protons) and AZD6738 compared with radiation alone at 2 hours after irradiation (Figure 4, E and M). At 4 hours after irradiation, we observed significantly lower 53BP1 foci numbers in NCI-H460 cells treated with radiation (photons or protons) combined with AZD6738 compared with radiation alone (Figure 4F). At 18 hours after irradiation, we observed significantly higher 53BP1 foci numbers in cells treated with radiation (photons or protons) and AZD6738 compared with radiation alone (Figure 4, G and O). Similar to γH2AX foci number kinetics, we found that 53BP1 foci number decreased as a function of time. 53BP1 foci resolved more slowly when cells were treated with radiation and AZD6738 compared with radiation alone. The slowest relative decrease in 53BP1 foci number as a function of time was observed for protons combined with AZD6738 (Figure 4, H and P).

### AZD6738, alone or in combination with radiation, increases the formation of MN.

**Figure 5:**
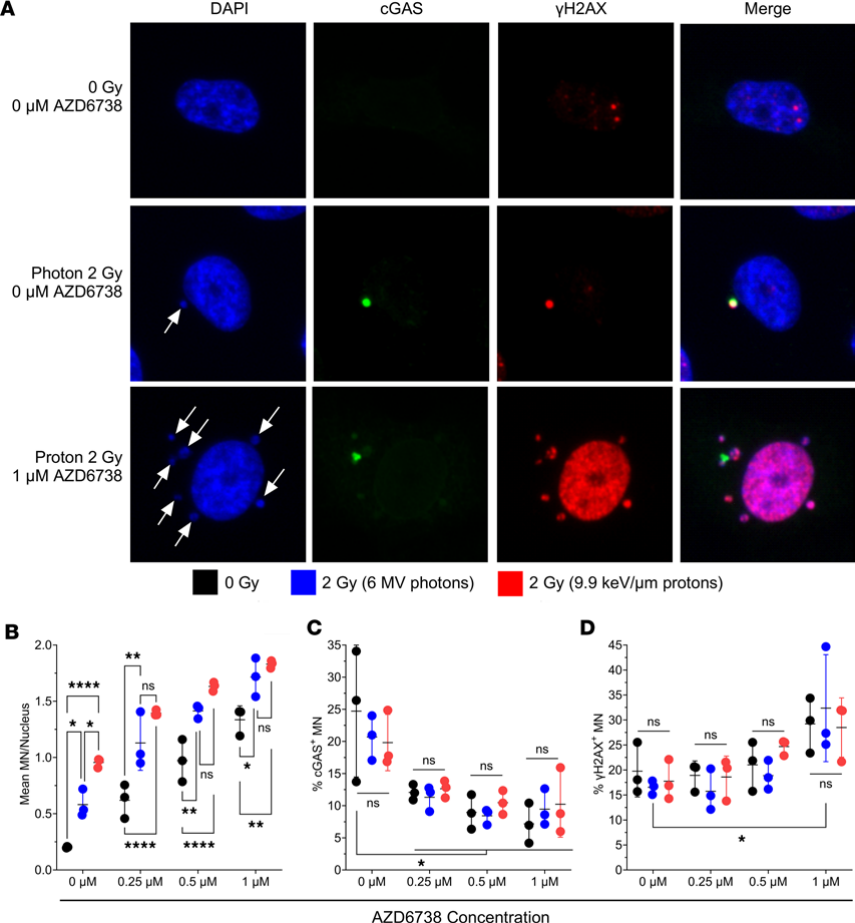
Radiation plus AZD6738 amplifies micronuclei (MN) numbers in NCI-H1299 cells 48 hours after irradiation. (**A**) Representative nuclei and MN (white arrows) colocalized with cGAS, γH2AX, neither, or both proteins. Original magnification, ×20. (**B**) Mean MN numbers per nucleus. (**C**) Percentages of MN colocalized with cGAS relative to total number of MN. (**D**) Percentages of MN colocalized with γH2AX relative to total number of MN. Error bars represent the SD from 3 independent experiments. Statistical significance was assessed with 2-way ANOVA with Tukey’s multiple-comparison test. NS, non-significant. *P < 0.05; **P < 0.01; ****P < 0.0001.

Having observed that AZD6738 delays the kinetics of γH2AX and 53BP1, potentially disrupting NHEJ, we studied the consequences of AZD6738 on MN formation. MN formation is linked to unrepaired DNA lesions, particularly in G_2_ and mitosis, and aberrant cell cycle control, so we next investigated whether combining AZD6738 with photons or protons would augment MN production. MN and cytoplasmic DNA, when recognized by cGAS, can stimulate antitumor immune signaling. We observed that both photons and protons alone increased MN formation (Figure 5, A and B), with protons inducing significantly greater numbers of MN than photons. The addition of AZD6738, at several concentrations, significantly increased MN formation alone and in combination with radiation (photons and protons). In vehicle-treated groups irrespective of radiation dose (0 or 2 Gy) and radiation type (photons or protons), the level of cGAS colocalization with MN was constant. However, AZD6738, independent of radiation dose (0 or 2 Gy) and radiation type (photons or protons), significantly reduced the colocalization of cGAS (Figure 5C), but this finding should be considered in the context that the absolute number of MN and cGAS-positive MN were increased. Moreover, this finding may also have been influenced by the time of testing (at 48 hours). Very few differences were noted in the colocalization of γH2AX foci with MN (Figure 5D).

### AZD6738 plus radiation delays tumor growth.

**Figure 6:**
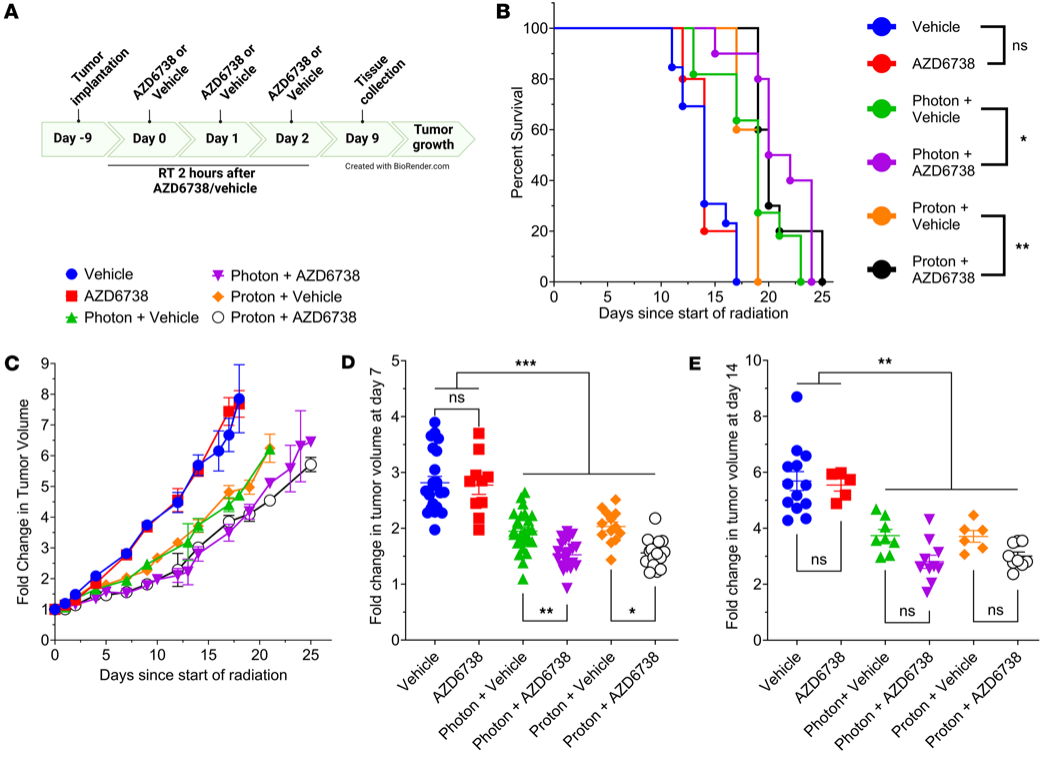
AZD6738 is an effective radiosensitizer in vivo. Radiation plus AD6738 effects on tumor growth and survival. (**A**) Timeline for treatments in BALB/c mice bearing 100–200 mm^3^ 4T1 tumors. (**B**) Survival, determined by quintupling of tumor size, after sham irradiation or irradiation with photons or protons combined with AZD6738 (75 mg/kg) or a vehicle control, given 2 hours before irradiation. Mice were treated with 18 Gy, given in three 6-Gy fractions delivered approximately 24 hours apart. (**C**) Corresponding tumor growth curve reported as fold change relative to tumor volume at day 0. (**D** and **E**) Fold change in tumor volume relative to tumor volume at day 0, at (**D**) 7 days, and (**E**) 14 days after the start of radiation treatment. In **D** and **E**, each symbol represents 1 mouse. Error bars represent the SD. Statistical significance was assessed with the log-rank Mantel-Cox test (**B**) or 1-way ANOVA with Tukey’s multiple-comparison test (**D** and **E**). NS, non-significant. *P < 0.05; **P < 0.01; ***P < 0.001.

The 4T1 cell line is an aggressive model of triple-negative breast cancer that is radiosensitized by AZD6738 to photons and protons in vitro (Figure 1 and Supplemental Figure 2). Mice bearing 4T1 tumors were sham irradiated or irradiated with photons or protons to a total dose of 18 Gy, given as three 6-Gy fractions delivered approximately 24 hours apart. Mice were also treated with 75 mg/kg AZD6738 or a vehicle agent as a control at 2 hours before each irradiation (Figure 6A). All irradiated groups, regardless of vehicle or AZD6738 treatment, showed significantly extended survival relative to unirradiated groups (Figure 6B). Mice treated with photons plus AZD6738 had significantly longer survival than did mice treated with photons alone, with the same pattern observed following proton treatment. It should be noted that the survival endpoint was defined as a 5-fold increase (relative to the first day of irradiation) in tumor volume and is presented to support tumor-growth-delay findings. In our setup, no significant difference was observed between photons and protons without AZD6738. Sham-irradiated groups treated with AZD6738 showed no difference in tumor growth versus the vehicle-alone group, but groups treated with photons or protons plus vehicle or AZD6738 showed significantly delayed tumor progression (Figure 6C). Calculations of tumor volume on day 7 (Figure 6D) and day 14 (Figure 6E) after irradiation showed that all irradiated groups had significantly smaller tumor volumes at both times. On day 14, the combination of photons plus AZD6738 led to significantly smaller tumors compared with photons alone. A nonsignificant difference was noted between proton plus vehicle or proton plus AZD6738 groups.

### AZD6738 modulates the immune response to radiotherapy.

**Figure 7:**
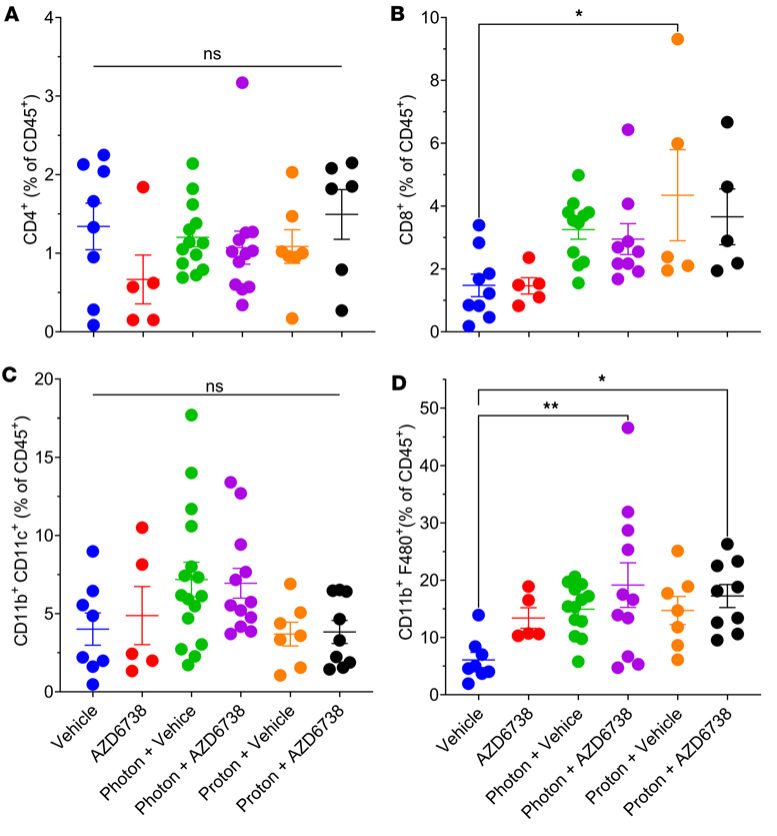
AZD6738 modulates the immune response to radiotherapy in the tumor microenvironment. Immune cell populations harvested at 9 days after the first treatment day. (**A**) CD4^+^ T cells. (**B**) CD8^+^ T cells. (**C**) Dendritic cells, defined as CD11b^+^CD11c^+^. (**D**) Macrophages, defined as CD11b^+^F480^+^. Each symbol represents 1 mouse. Error bars represent the SEM. Statistical significance was assessed with 1-way ANOVA with Tukey’s multiple-comparison test. NS, non-significant. *P < 0.05; **P < 0.01.

As an initial evaluation of the effects of AZD6738 and radiation on the immune response, we also investigated the infiltration of various immune populations, including CD4^+^ and CD8^+^ T cells, dendritic cells (CD11b^+^CD11c^+^), and macrophages (CD11b^+^F480^+^) into the tumor. We observed very little difference in CD4^+^ T cell infiltration (Figure 7A), although CD8^+^ T cell infiltration seemed to increase in all irradiated groups. This increase was statistically significant only after proton irradiation (Figure 7B). Our examination of myeloid populations such as dendritic cells and macrophages revealed no differences in dendritic cells in any group (Figure 7C), but AZD6738 plus either type of radiation led to greater numbers of macrophages compared with vehicle alone, whereas radiation alone led to modest increases (Figure 7D). No differences were observed between photons and protons with or without AZD6738 (Figure 7D).

### AZD6738 plus radiation modulates functional subsets of immune populations.

**Figure 8:**
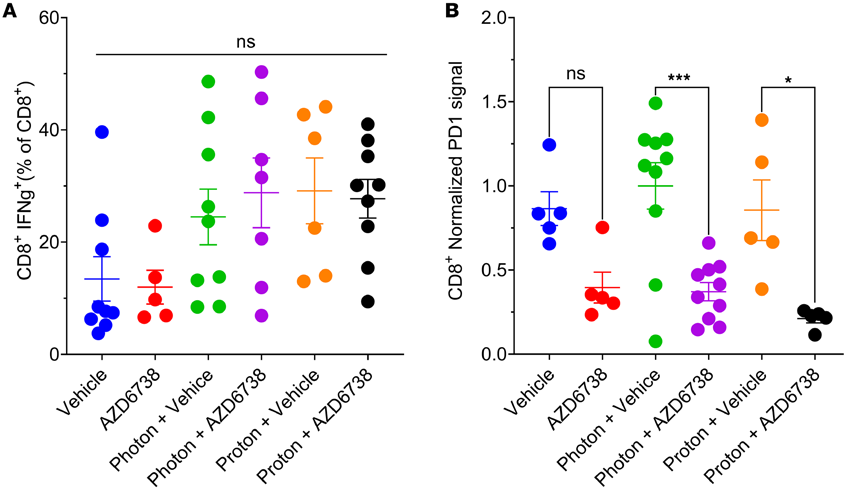
AZD6738 plus radiation modulates functional subsets of immune populations in the tumor microenvironment. Characterization of immune cell populations (including IFN-γ^+^CD8^+^ T cells and PD-1–expressing CD8^+^ T cells) harvested 9 days after the first treatment day. (**A**) IFN-γ^+^CD8^+^ T cell populations after irradiation plus AZD6738. (**B**) PD-1–expressing CD8^+^ T cell populations after irradiation plus AZD6738. Error bars represent the SEM. Statistical significance was assessed with 1-way ANOVA with Tukey’s multiple-comparison test. NS, non-significant. *P < 0.05; ***P < 0.001.

We next investigated whether AZD6738, in combination with photons or protons, modulates specific subsets of immune cells. We examined CD8^+^ T cell activation by measuring interferon-γ (IFN-γ) levels in CD8^+^ T cells and examined the expression of PD-1 on CD8^+^ T cells. All irradiated groups showed modest (but not significant) increases in IFN-γ in CD8^+^ T cells (Figure 8A), and AZD6738 treatment significantly reduced the expression of PD-1 on CD8^+^ T cells with or without radiation (Figure 8B).

## Discussion

A therapeutic opportunity exists to improve radiotherapy through the combination of radiation modalities that produce more compact dose distributions, sparing more normal tissue, and small molecule inhibitors that are potent radiosensitizers. We have demonstrated that when looking at protons in isolation, cancer cells and HUVEC could be effectively radiosensitized by ATR inhibition, with increases in the sensitization enhancement ratio (SER) at SF2Gy. However, it should be noted that the radiosensitization depended on the concentration of the ATR inhibitor and did not necessarily lead to an increased RBE. We also showed that in some cases ATR inhibition increased the RBE of proton therapy. While not explored here, it will be important to understand why some cell lines showed increased RBE, while others were unchanged. Zhou et al. recently demonstrated a mechanistic basis for modulation of proton RBE that is reliant on the ligation step associated with NHEJ, with little reliance on RAD51 and HR (30). Zhou et al. were also able to increase proton RBE with an inhibitor of ATM, but not ATR. It is also possible that the concentrations of AZD6738 used by Zhou et al. radiosensitized cells irrespective of LET, and that concentrations could be fine-tuned to promote LET-dependent effects. An RBE increase after ATM, but not ATR, inhibition could be cell line specific, which is supported by our data for ATR inhibition. One of the limitations of our work is that most of our in vitro results on RBE were obtained with a high-LET proton condition (9.9 keV/μm). Although we observed an increased RBE after ATR inhibition for a LET that is typically found within tumors (3.85 keV/μm), lower LET is expected to reduce damage clustering, perhaps increasing RBE because for high LET, the RBE-enhancing effect of ATR inhibition may be lost as the damage induced by high-LET protons results in cell death regardless of ATR inhibition. We believe the radiosensitization induced by ATR inhibition is attributable to an increased amount of unresolved DNA damage and abrogation of the G_2_ cell cycle checkpoint. The combination of increased DNA damage and unhindered cell cycle progression in the presence of DNA damage led to increased MN formation, which is known to be immunostimulatory. To rule out off-target effects of a particular ATR inhibitor, we confirmed the effects of ATR inhibition using a different ATR inhibitor in our lung cancer models for clonogenic cell survival and RAD51 foci formation. We showed that both photons and protons were effective at delaying tumor growth when combined with ATR inhibition on day 7 after irradiation. All groups exposed to radiation showed significantly slower growth when assessed on day 14, although there was no significant difference between radiation and radiation plus AZD6738. Both photons and protons combined with ATR inhibition increased survival and led to changes in the immune microenvironment.

DNA repair inhibitors have gained attention as potentially synergistic additions to radiotherapy (13, 31, 32). Here we targeted the protein ATR because of its dual role in cell cycle arrest and in DNA repair (1, 33). We and others have shown that inhibition of ATR can lead to radiosensitization to photons (5, 7, 9, 10), protons (7), and carbon ions (34). The cell lines investigated here were all radiosensitized by ATR inhibition. PANC-1 was the most radioresistant cell line, with significant but modest radiosensitization by ATR inhibition, whereas Panc 10.05 showed much greater radiosensitization by ATR inhibition. The differences in degrees of radiosensitization suggest that different genotypes are an important consideration when combining DNA repair inhibitors with radiation of any form. Three (HUVEC, NCI-H460, and Panc 10.05) of the 7 cell lines tested showed increased RBE. HUVEC and NCI-H460 showed the biggest increases, especially at 1 and 2 μM AZD6738 concentrations. Notably, these were the only *TP53* wild-type cell lines investigated. Others have shown that *TP53* loss enhances sensitivity to ATR inhibition (11), although radiosensitization can also be achieved by disrupting ATR signaling regardless of *TP53* status (5, 35). The increased RBE could stem from the role of p53 in inducing apoptosis (36, 37). We also saw that the degree of radiosensitization by ATR inhibition was significant in HUVEC, which we used as an in vitro model of normal cells. Because ATR inhibition radiosensitizes HUVEC, proton therapy, which limits normal tissue exposure because of its physical characteristics, may be an appropriate radiation modality to combine with ATR inhibition. Our findings underscore the importance of understanding the potential for normal tissue radiosensitization, particularly in organs at risk, when combining radiotherapy with radiosensitizers such as ATR inhibitors.

We also explored potential mechanisms of AZD6738 radiosensitization by examining its effects on cell cycle and DNA damage signaling and repair. AZD6738 reduced the number of cells in G_2_ in both NCI-H460 and NCI-H1299 lung cancer cells following photons, and significantly increased the fraction of cells in mitosis after photons and protons, suggesting that ATR is critical for preventing the G_2_-M transition after DNA damage induced by both types of radiation used in this study. DNA damage was also significantly increased after AZD6738 plus radiation. This finding likely reflects a combination of replicative stress from unregulated cell cycle progression, which ATR usually helps to resolve (38), and defective HR, which has been observed after ATR inhibition (4, 39).

We used RAD51 foci as a surrogate to assess HR repair and identified that proton irradiation induced more RAD51 foci at 2 hours after irradiation in both NCI-H460 and NCI-H1299 cells, which supports the hypothesis that repair via HR is of greater importance for higher-LET radiation than for low-LET photons (17, 18, 20, 22, 40). We also observed that AZD6738 significantly reduced the number of RAD51 foci in NCI-H460 cells, but not in NCI-H1299 cells. ATR is known to promote HR, in part through inhibiting cyclin-dependent kinases and in part by directly phosphorylating PALB2 (41–43). These effects may depend on the cell cycle stage, and ATR may preferentially modulate HR in S phase, owing to its role in replicative stress (1, 33). Thus, differences may not be observed in the analysis here, which was independent of cell cycle stage.

ATR inhibition delayed the kinetics of γH2AX and 53BP1 recruitment after radiation-induced DNA DSB damage, indicating that DNA repair is impaired. Protons in combination with ATR inhibition showed the slowest resolution of γH2AX and 53BP1 foci, likely due to the higher yield of clustered DNA damage induced by protons versus photons. γH2AX is important for DNA damage signal amplification and recruitment of DNA repair factors such as the pro-NHEJ 53BP1 (44–46). While 53BP1 is not strictly necessary for NHEJ to proceed, it prevents DNA end resection, promoting repair through NHEJ (47), as opposed to HR. Our data indicate that ATR inhibition reduced γH2AX foci 2 hours after irradiation. This could explain the reduced radiation-induced 53BP1 foci after ATR inhibition compared with radiation alone at the early time points (2 and 4 hours after irradiation), indicating that NHEJ is delayed after ATR inhibition. The numbers of persistent γH2AX and 53BP1 foci at 18 hours after irradiation were significantly higher in the groups treated with AZD6738 compared with radiation alone, which could be due to lesions that would ordinarily be repaired by HR (which is inhibited under ATR inhibition) having to utilize NHEJ machinery, which is either ineffective or significantly slower at repairing the lesion in question.

In addition to the radiosensitization effect of AZD6738, we explored its ability to augment radiation-induced formation of MN, an important component of radiation-induced antitumor immunity (48, 49). We observed that the number of MN per nucleus was significantly increased with protons relative to photons, which is likely attributable to the increased damage clustering associated with high-LET protons, indicated by the slower kinetics of γH2AX and 53BP1 foci disappearance as a function of time for protons combined with ATR inhibition. In the presence of AZD6738, the number of MN per nuclei was increased for photons and protons and for AZD6738 alone. The disruption in cell cycle checkpoint blockade and increased residual DNA damage induced by the combination of AZD6738 and radiation is likely responsible for the increased MN count. In addition to the number of MN, we assessed MN that colocalized with the immunostimulatory protein cGAS and the DNA damage response protein γH2AX. Although little difference was seen in colocalization of γH2AX regardless of radiation or AZD6738 treatment, AZD6738 did seem to reduce the percentage of cGAS-colocalized MN. Notably, however, the absolute number of cGAS-positive MN was increased. The activation of cGAS has been reported to be influenced by several factors, including fragmented DNA length. Specifically, DNA fragments smaller than 40 base pairs did not activate cGAS (50, 51), but cGAS potency increased as the DNA length extended up to 4003 base pairs, which would allow cGAS dimerization and result in robust activation (52). When MN are formed, DNA-associated proteins such as histones and DNA repair proteins can be included in the MN. Recent findings suggest that the specific protein contents sequestered within MN can profoundly affect the likelihood of successful cGAS binding. Abdisalaam et al. (53) demonstrated that recruitment of Nijmegen breakage syndrome 1 to MN led to carboxy-terminal binding protein 1 interacting protein–dependent DNA resection, rendering cGAS unable to effectively bind. Others have shown that the chromatin state of the DNA within MN is an important determinant of cGAS binding, with histone 3 lysine 79 demethylation regulating cGAS recruitment (54). How ATR inhibition affects MN DNA organization is unclear, but may be a relevant consideration, particularly with regard to timing relative to radiation and may explain why the percentage of cGAS-positive MN was reduced. The temporal aspects of cGAS binding were not investigated here, and may offer more insight as to why the positive percentages were reduced at 48 hours after irradiation.

We used the triple-negative breast cancer cell line 4T1 to determine the effects of AZD6738 in vivo in BALB/c mice, including effects on immune activation, which has been reported with radiation (49, 55, 56). We observed very little effect with AZD6738 alone as a monotherapy; however, tumor growth could be significantly delayed with radiation. In combination treatments, photons with AZD6738 showed delay in tumor growth on day 14 and could significantly prolong survival compared with photons alone. Although not significant, protons alone compared to combined with AZD6738 showed the same trending differences as photons.

Based on our in vitro data, which showed increased MN accumulation with the potential for antitumor immune signaling, we were interested in whether AZD6738 combined with radiation could stimulate the recruitment of antitumor immune populations to the primary lesion. We used the 4T1 model, which is aggressive and poorly immunogenic. In our system, no differences were observed between sham irradiated and mice treated with AZD6738 alone. We also did not see any differences in the infiltration of CD4^+^ T cells in any irradiated groups. However, CD8^+^ T cell infiltration was increased in all irradiated groups, with significant increases in the proton plus vehicle group versus vehicle control. AZD6738 did not modulate this effect with either radiation type. Protons also seemed to increase the number of CD8^+^ T cells relative to photons, although this was not significant. To determine the functional state of these CD8^+^ T cells, we looked at levels of IFN-γ, which increased with radiation independently of AZD6738. Immune cells can also quickly become overwhelmed by the immunosuppressive environment within the tumor. We therefore investigated PD-1 expression on CD8^+^ T cells. We found that AZD6738 could reduce the expression of PD-1 on CD8^+^ T cells, which has also been observed by others (27, 28). Interest has also been expressed in combining radiotherapy with immune checkpoint inhibitors; in that regard, our results raise important questions as to which checkpoint to target if PD-1 is already being reduced by ATR inhibition in CD8^+^ T cells and photon and proton radiation do not overcome this reduction.

In addition, both photons and protons in combination with AZD6738 increased CD11b^+^F480^+^ macrophages. Although we did not explore the specific mechanism of this effect, it probably resulted from cell death processes and immunogenic signaling. Macrophages, depending on their polarization, can have protumorigenic or potent antitumor functions.

The effects of ATR inhibition on the whole immune cell population (not just those cells located in the tumor) should also be considered. Sugatini et al. (57) reported that ATR inhibition in activated CD8^+^ T cells can be cytotoxic due to the toxic buildup of deoxyuridine in genomic DNA. Furthermore, Vendetti et al. (58) demonstrated that ATR scheduling when combined with radiation can have profound effects on the immune response in the tumor and draining lymph nodes, with prolonged ATR inhibition preventing the expansion of activated T cells. Similar results were also observed by Hardakker et al. (59), who also noted that ATR inhibition can induce antitumor immune effects directly on immune cells without an exogenous DNA damaging agent applied to the tumor when using an intermittent schedule of ATR inhibition (7 days on, 7 days off). Our study used a short course schedule of ATR inhibition delivered for a total of 3 days concomitant with radiation. While this should allow recovery of activated T cells, it did not necessarily maintain immune-activating effects associated with an on/off schedule, particularly in the production of IFN type I. We note that the scheduling of ATR inhibition, or any other DNA damage repair inhibitor in general, with radiation is an incredibly important factor in determining the response being measured.

While our study offers insight into the potential of using AZD6738 as a radiosensitizer for photons and protons, a limitation of our study is that most of our in vitro assays used an unmodulated proton beam with a dose-weighted LET of 9.9 keV/μm, while our in vivo studies used spread-out Bragg peak parallel opposed beams, which resulted in a dose-weighted LET of 3.99 keV/μm. We also acknowledge that without a more comprehensive analysis of the immune populations assayed, it is hard to confirm whether this is a robust antitumor immune response. Our study used previously reported concentrations of AZD6738 in combination with radiation (5, 27, 28), which have been shown to reach levels of 10.1 μg/mL in tumor tissue (60). However, we did not perform our own pharmacodynamic studies to confirm an efficacious AZD6738 concentration in the tumor.

In summary, we have shown that inhibition of ATR is an effective strategy for radiosensitization in vitro and in some cases can increase the RBE of proton radiotherapy, possibly in p53-proficient cells, findings that have implications for normal tissue toxicity. ATR inhibition leads to radiosensitization by abrogating the G_2_ checkpoint, allowing cells unrestricted access into mitosis in addition to disrupting DNA repair, at least in part through reduced HR. ATR inhibition in combination with photons or protons also increased MN numbers, which may induce immune signaling. We also noted that ATR inhibition in combination with radiation was effective in delaying 4T1 tumor growth and extending survival in vivo. ATR inhibition combined with radiation also modulated the myeloid populations in tumors, including macrophages, which could have pro- or antitumorigenic effects, as well as downregulating PD-1 expression. Understanding the function of these populations in more detail and how they can be modulated may yield further strategies to increase antitumor immunity and better patient outcomes.

## Methods

Further experimental details and reagents can be found in the Supplemental Methods and Supplemental Table 4.

### Sex as a biological variable.

We used only female mice in our studies because breast cancer is prevalent in this sex.

### Cell lines.

NCI-H1299 and NCI-H460 were purchased from American Type Culture Collection and cultured in RPMI-1640 (Sigma-Aldrich, R8758) supplemented with 10% fetal bovine serum (FBS) (Sigma-Aldrich, F0926) and 1% penicillin-streptomycin (P/S) (Hyclone, SV30010). MDA-MB-231 (a gift from Dadi Jiang, The University of Texas MD Anderson Cancer Center) was cultured in RPMI-1640 supplemented with 10% FBS and 1% P/S. PANC-1 and Panc 10.05 (purchased from American Type Culture Collection) were grown in DMEM (Sigma-Aldrich, D6429) supplemented with 10% FBS and 1% P/S. 4T1 (a gift from Asaithamby Aroumougame, The University of Texas Southwestern Medical Center, Dallas, Texas, USA) was cultured in RPMI-1640 supplemented with 10% FBS and 1% P/S. Human and mouse cell lines were authenticated by using short tandem repeat markers at the MD Anderson Cytogenetics and Cell Authentication Core facility and IDEXX BioAnalytics, respectively. The HUVEC line (a gift from Keri Schadler, The University of Texas MD Anderson Cancer Center) were cultured in endothelial cell medium (ScienCell, 1001) supplemented with 5% FBS, 1% P/S, and 1% endothelial cell growth supplement. HUVEC is a nontransformed endothelial cell line; for simplicity they are referred to as “normal cells.” All cell lines were maintained in a humidified incubator with 5% CO_2_ at 37°C. All cell lines were confirmed to be free of mycoplasma contamination at the MD Anderson Cytogenetics and Cell Authentication Core facility before, during, and after experiments.

### In vitro treatments.

Cells were irradiated with photons or protons as described previously (17) (Supplemental Method 1 and Supplemental Figure 6). Briefly, photons were delivered by a 6-MV clinical linear accelerator (Truebeam, Varian Medical Systems) at a water equivalent depth of 10 cm. Protons were delivered at the MD Anderson Proton Therapy Center with an unmodulated proton beam (4.3 cm range in water) at a water equivalent depth of 4.42 cm, with a dose-weighted LET in water of 9.9 keV/μm. We have also performed limited irradiations using a modulated proton beam (16.5 cm range in water and spread-out Bragg peak of 4 cm) at a water equivalent depth of 15.6 cm, with a dose-weighted LET in water of 3.85 keV/μm. Most of the proton irradiations for our in vitro irradiations were done with high LET relative to what is typically found clinically within the tumor volume. This high-LET condition was chosen to maximize the physical differences to photons. The LET was determined with a validated Monte Carlo model of the proton beam nozzle (61). AZD6738 (at 10 mM in DMSO) was purchased from Selleckchem; aliquots were prepared and stored at –80°C until use.

### Clonogenic survival.

Clonogenic assays were performed as described previously (17). Briefly, cells were seeded into 6-well plates. The original medium was removed 6–8 hours before irradiation and replaced with complete medium supplemented with AZD6738. DMSO was used as a control (vehicle). Total incubation time with each inhibitor was 24 hours (6–8 hours before and 16–18 hours after irradiation), after which medium containing inhibitor was replaced with fresh medium. Clonogenic plates were then incubated for 7–14 days, after which colonies were stained with 0.5% crystal violet (HT90132, Sigma-Aldrich) in 100% ethanol. Plates were air-dried overnight and scanned with a high-resolution flatbed scanner (Epson Expression 10000 XL). Colonies were scored by using custom-built ImageJ macros that were tailored to each cell line and optimized for colonies of 50 or more cells (17). At least 3 biological repeats were performed for each condition. Each biological repeat contained at least 2 replicates.

### Immunofluorescence.

Cells were fixed with 4% paraformaldehyde and washed with PBS 3 times (10 minutes per wash). Cells were permeabilized with 0.3% Triton X-100 in PBS for 10 minutes at room temperature. Cells were blocked with 5% goat serum, 0.2% fish gelatin, and 0.1% Tween 20. Cells were incubated with primary antibodies in blocking solution (described above) overnight in a humidity chamber. Cells were washed 3 times with PBS (10 minutes per wash) before being stained with secondary antibodies in blocking solution for 1 hour at room temperature. Nuclei were counterstained with DAPI before being mounted with Fluoromount G. Foci were analyzed with CellProfiler (62), and MN were counted with ImageJ (NIH).

### Cell cycle and mitotic index.

Cells were trypsinized and stained with FXcycle according to the manufacturer’s instructions. Briefly, cells were centrifuged at 4°C for 5 minutes at 300*g* and washed with PBS twice before being fixed in 70% ice-cold ethanol and stored at –20°C. Ethanol was added with constant agitation. Before being analyzed, cells were centrifuged at room temperature for 5 minutes at 400*g* and washed with PBS; this was done twice. Cells were then incubated with anti-H3S10ph (D2C8) antibody conjugated to Alexa Fluor 488 for 30 minutes at room temperature in PBS supplemented with 2% FBS. Cells were washed 3 times with PBS by centrifugation at 400*g* for 5 minutes and resuspended in PBS before the samples were analyzed with a flow cytometer (Attune NxT, Thermo Fisher Scientific).

### In vivo treatment and processing.

4T1 cells (5 × 10^4^) triple-negative breast cancer cells were injected in the left lower leg of female BALB/c mice 7–9 days before irradiation. Mice bearing tumors between 150 and 400 mm^3^ were included in the analysis. ATR inhibitor (75 mg/kg, AZD6738) was given 2 hours before irradiation via intraperitoneal injection; mice were irradiated to a total dose of 18 Gy, given in three 6-Gy fractions with 6-MV photons or protons with a dose-weighted LET in water of 3.99 keV/μm (Supplemental Method 1 and Supplemental Figure 6). Tumor volumes were measured manually with calipers and volume calculated as *a*^2^ × *b*/2, where *a* and *b* are the shortest and longest dimensions, respectively. Survival was defined as the duration of time for tumors to increase in size 5-fold relative to the tumor size on the first day of irradiation. Tumor-infiltrating lymphocytes were processed as described in Supplemental Method 2 and Supplemental Figure 7. Briefly, tumors were excised and dissociated with scalpels and Liberase in RPMI media supplemented with DNase. Cells were collected and isolated using a lymphocyte separation solution. Cells were incubated with TruStain FcX Plus, following staining with an antibody cocktail, and then fixed with paraformaldehyde. If intracellular staining was required, cells were permeabilized and incubated with appropriate labelled antibodies. Cells were analyzed at MD Anderson’s Flow Cytometry and Cellular Imaging core facility.

### Data analysis.

Clonogenic data were analyzed as described previously (7, 17). Briefly, various metrics were calculated from survival curves (Supplemental Method 3 and Supplemental Figure 8) and compared to determine the SER and the RBE:

SER_r,i_ (*M*) = *M*_r,vehicle_/*M*_r,AZD6738_ Eq. 1

RBE_r_ (*M*) = *M*_x-ray,i_/*M*_proton,i_ Eq. 2

where *M* is a given metric from the survival curve, *i* is the vehicle or AZD6738, and *r* is the radiation type.

### Statistics.

The data were analyzed using Prism (version 10, GraphPad). Data are expressed as mean ± standard deviation (SD) or mean ± standard error of the mean (SEM). The Gaussian distribution of the data was investigated by D’Agostino-Pearson or Shapiro-Wilk tests to determine the normality of the data prior selecting the appropriate statistical test to perform. For comparisons among more than 2 groups, statistical differences in parametric data were assessed using 1-way analysis of variance (ANOVA) with Tukey’s multiple-comparison test or 2-way ANOVA with Tukey’s multiple-comparison test. Unpaired *t* test (parametric) was used to compare 2 groups (supplemental data). Survival in vivo was assessed by using the log-rank Mantel-Cox test. *P* values of less than 0.05 were considered to indicate significant differences.

### Study approval.

All animal manipulations and procedures, including tumor implantation, irradiation, and drug treatment were done under a protocol (1590-RN02) approved by The University of Texas MD Anderson Cancer Center Institutional Animal Care and Use Committee.

### Data availability.

All data generated and analyzed during this study are included in the published article and its supplemental information files, including values for all data points shown in graphs and values behind any reported means in the supplemental Supporting Data Values file. Key resources used in the study can be found in the supplemental information.

## Author contributions

SJB conceptualized the study, acquired and analyzed data, generated figures, prepared the manuscript, and supervised the study. MM, DBF, RK, and MBK acquired and analyzed data. DKJM and CHM acquired data. IQ and BXT acquired and analyzed data, and reviewed and edited the manuscript. PCM analyzed data, and reviewed and edited the manuscript. SFS conceptualized the study, interpreted data, reviewed and edited the manuscript, acquired funding, and supervised the study. GOS conceptualized the study, acquired and interpreted data, reviewed and edited the manuscript, acquired funding, and supervised the study.

## Supplementary Material

Supplemental data

Supplemental tables 1-3

Supporting data values

## Version 1 09/05/2024

In-Press Preview

## Version 2 10/08/2024

Electronic publication
