# Supplementary material for: ATR inhibition radiosensitizes cells through augmented DNA damage and G2 cell cycle arrest abrogation
Source: JCI Insight. 2024 Oct 8;9(19):e179599. doi: 10.1172/jci.insight.179599 (PMC11466186; doi:10.1172/jci.insight.179599)
Supplement: Supplemental data [file jciinsight-9-179599-s098.pdf]

Supplementary Material: ATR inhibition radiosensitizes cells through augmented DNA Damage and G2 cell cycle arrest abrogation
Bright et al.

Supplementary Table 1**.** Survival curve parameters for in vitro clonogenic assays.

Cell Line	Radiation Type	AZD6738 Concentration (μM)	n	α (Gy^{-1})	σ_{α} (Gy^{-1})	β (Gy^{-2})	σ_{β} (Gy^{-2})	Covariance
HUVEC	6 MV	0	4	0.328	0.034	0.0085	0.0052	-0.9671
HUVEC	6 MV	0.1	5	0.393	0.034	0.0082	0.0049	-0.9648
HUVEC	6 MV	2	7	0.611	0.085	0.0538	0.0249	-0.9282
HUVEC	9.9 keV/μm	0	4	0.636	0.085	0.0120	0.0168	-0.9563
HUVEC	9.9 keV/μm	0.1	4	0.656	0.060	0.0171	0.0145	-0.9548
HUVEC	9.9 keV/μm	2	4	1.215	0.133	0.0000	0.0539	-0.9531
H460	6 MV	0	11	0.227	0.041	0.0612	0.0090	-0.9624
H460	6 MV	0.1	6	0.269	0.053	0.0963	0.0128	-0.9602
H460	6 MV	1	4	0.772	0.093	0.0000	0.0237	-0.939
H460	9.9 keV/μm	0	11	0.310	0.117	0.1204	0.0362	-0.9644
H460	9.9 keV/μm	0.1	7	0.526	0.082	0.1498	0.0254	-0.9645
H460	9.9 keV/μm	1	4	1.445	0.207	0.0000	0.0000	-0.9494
H1299	6 MV	0	11	0.092	0.038	0.0219	0.0050	-0.971
H1299	6 MV	0.1	5	0.259	0.032	0.0040	0.0051	-0.9547
H1299	6 MV	1	4	0.595	0.084	0.0081	0.0144	-0.9463
H1299	9.9 keV/μm	0	9	0.226	0.045	0.0205	0.0077	-0.9661
H1299	9.9 keV/μm	0.1	5	0.100	0.028	0.0521	0.0052	-0.959
H1299	9.9 keV/μm	1	4	0.745	0.134	0.0102	0.0311	-0.9491
PANC1	6 MV	0	5	0.182	0.038	0.0297	0.0058	-0.9636
PANC1	6 MV	0.1	4	0.143	0.038	0.0362	0.0058	-0.9629
PANC1	6 MV	2	4	0.263	0.051	0.0354	0.0097	-0.9383
PANC1	9.9 keV/μm	0	5	0.302	0.039	0.0373	0.0084	-0.9599
PANC1	9.9 keV/μm	0.1	5	0.201	0.068	0.0767	0.0153	-0.9565
PANC1	9.9 keV/μm	2	5	0.356	0.065	0.0688	0.0182	-0.951
PANC10.05	6 MV	0	4	0.214	0.050	0.0129	0.0066	-0.9645
PANC10.05	6 MV	0.1	3	0.269	0.071	0.0228	0.0095	-0.9561
PANC10.05	6 MV	2	4	0.468	0.132	0.0518	0.0335	-0.9454
PANC10.05	9.9 keV/μm	0	5	0.274	0.065	0.0319	0.0110	-0.9674
PANC10.05	9.9 keV/μm	0.1	5	0.202	0.052	0.0673	0.0099	-0.9522
PANC10.05	9.9 keV/μm	2	5	0.885	0.124	0.0048	0.0369	-0.9633
MDA-MB-231	6 MV	0	8	0.307	0.056	0.0271	0.0087	-0.9596
MDA-MB-231	6 MV	0.1	6	0.363	0.048	0.0248	0.0077	-0.9596
MDA-MB-231	6 MV	2	6	0.685	0.090	0.0122	0.0154	-0.9433
MDA-MB-231	9.9 keV/μm	0	5	0.614	0.055	0.0373	0.0130	-0.9505
MDA-MB-231	9.9 keV/μm	0.1	4	0.810	0.080	0.0035	0.0171	-0.9576
MDA-MB-231	9.9 keV/μm	2	4	1.087	0.119	0.0000		-0.9425
4T1	6MV	0	9	0.103	0.033	0.0291	0.0047	-0.9731
4T1	6MV	0.1	6	0.059	0.023	0.0351	0.0033	-0.9726
4T1	6MV	0.5	7	0.315	0.061	0.0173	0.0089	-0.9594
4T1	3.85 keV/μm	0	3	0.026	0.045	0.0498	0.0071	-0.9783
4T1	3.85 keV/μm	0.5	3	0.000	0.301	0.1750	0.0587	-0.9433
4T1	9.9 keV/μm	0	4	0.158	0.115	0.0707	0.0238	-0.9583
4T1	9.9 keV/μm	0.1	4	0.108	0.074	0.0732	0.0150	-0.9608
4T1	9.9 keV/μm	0.5	4	0.275	0.123	0.0832	0.0267	-0.9565

Supplementary Table 2. Survival metrics calculated from the linear quadratic model.

Cell Line	Radiation Type	AZD6738 Concentration (μM)	SF2Gy	σ_{SF2Gy}	$D_{50\%}$ (Gy)	$\sigma_{D50\%}$ (Gy)	$D_{10\%}$ (Gy)	$\sigma_{D10\%}$ (Gy)
HUVEC	6 MV	0	0.5013	0.0244	2.0073	0.1349	6.0643	0.1250
HUVEC	6 MV	0.1	0.4412	0.0215	1.7041	0.1042	5.2805	0.1210
HUVEC	6 MV	2	0.2374	0.0206	1.0387	0.0893	2.9827	0.1030
HUVEC	9.9 keV/μm	0	0.2674	0.0287	1.0692	0.1100	3.4049	0.1641
HUVEC	9.9 keV/μm	0.1	0.2513	0.0170	1.0285	0.0689	3.2356	0.0880
HUVEC	9.9 keV/μm	2	0.0880	0.0078	0.5705	0.0624	1.8951	0.2071
H460	6 MV	0	0.4971	0.0242	1.9877	0.1035	4.5527	0.0656
H460	6 MV	0.1	0.3970	0.0234	1.6269	0.0940	3.6875	0.0577
H460	6 MV	1	0.2137	0.0218	0.8984	0.1080	2.9846	0.3586
H460	9.9 keV/μm	0	0.3321	0.0337	1.4349	0.1490	3.2704	0.0936
H460	9.9 keV/μm	0.1	0.1917	0.0136	1.0207	0.0702	2.5397	0.0512
H460	9.9 keV/μm	1	0.0556	0.0230	0.4729	0.0674	1.5764	0.2255
H1299	6 MV	0	0.7623	0.0437	3.9054	0.2942	8.3654	0.1903
H1299	6 MV	0.1	0.5861	0.0268	2.5724	0.1864	7.9137	0.3252
H1299	6 MV	1	0.2947	0.0339	1.1475	0.1283	3.6869	0.2137
H1299	9.9 keV/μm	0	0.5867	0.0358	2.5031	0.2061	6.4411	0.1724
H1299	9.9 keV/μm	0.1	0.6646	0.0238	2.8114	0.1017	5.7568	0.0702
H1299	9.9 keV/μm	1	0.2165	0.0335	0.9192	0.1290	2.9706	0.2014
PANC1	6 MV	0	0.6169	0.0334	2.6558	0.1846	6.2588	0.1151
PANC1	6 MV	0.1	0.6502	0.0357	2.8270	0.1876	6.2420	0.1092
PANC1	6 MV	2	0.5131	0.0347	2.0637	0.1680	5.1659	0.1464
PANC1	9.9 keV/μm	0	0.4713	0.0224	1.8676	0.1050	4.7943	0.0822
PANC1	9.9 keV/μm	0.1	0.4922	0.0395	1.9687	0.1587	4.3224	0.0997
PANC1	9.9 keV/μm	2	0.3724	0.0244	1.5069	0.1075	3.7486	0.0908
PANC10.05	6 MV	0	0.6185	0.0458	2.7710	0.3127	7.4237	0.2410
PANC10.05	6 MV	0.1	0.5330	0.0566	2.1752	0.3047	5.7539	0.2668
PANC10.05	6 MV	2	0.3188	0.0460	1.2953	0.1984	3.5364	0.1847
PANC10.05	9.9 keV/μm	0	0.5085	0.0445	2.0416	0.2185	5.2221	0.1476
PANC10.05	9.9 keV/μm	0.1	0.5104	0.0345	2.0434	0.1432	4.5396	0.0926
PANC10.05	9.9 keV/μm	2	0.1670	0.0188	0.7797	0.0841	2.5655	0.1166
MDA-MB-231	6 MV	0	0.4854	0.0382	1.9282	0.1870	5.1539	0.1573
MDA-MB-231	6 MV	0.1	0.4382	0.0297	1.7101	0.1372	4.7832	0.1333
MDA-MB-231	6 MV	2	0.2419	0.0300	0.9941	0.1065	3.1809	0.1958
MDA-MB-231	9.9 keV/μm	0	0.2522	0.0159	1.0603	0.0646	3.1475	0.0765
MDA-MB-231	9.9 keV/μm	0.1	0.1951	0.0188	0.8524	0.0692	2.8078	0.1243
MDA-MB-231	9.9 keV/μm	2	0.1137	0.0271	0.6306	0.0698	2.1169	0.2322
4T1	6 MV	0	0.7242	0.0340	3.4202	0.1959	7.2992	0.1100
4T1	6 MV	0.1	0.7725	0.0254	3.6840	0.1306	7.3049	0.0732
4T1	6 MV	0.5	0.4967	0.0435	1.9828	0.2267	5.5876	0.2088
4T1	3.85 keV/μm	0	0.7786	0.0483	3.4841	0.1978	6.5503	0.0940
4T1	3.85 keV/μm	0.5	0.4966	0.1927	1.9902	0.5557	3.6273	0.3497
4T1	9.9 keV/μm	0	0.5492	0.0778	2.2059	0.3121	4.6959	0.1882
4T1	9.9 keV/μm	0.1	0.6017	0.0557	2.4289	0.2129	4.9218	0.1232
4T1	9.9 keV/μm	0.5	0.4139	0.0609	1.6745	0.2459	3.8631	0.1628

Supplementary Table 3. Proton relative biological effectiveness in the indicated cell lines.

Cell Line	Radiation Type	AZD6738 Concentration (μM)	$RBE_{D10\%}$	$\sigma_{RBED10\%}$	$RBE_{D50\%}$	$\sigma_{RBED50\%}$
HUVEC	9.9 keV/μm	0	1.78	0.09	1.88	0.40
HUVEC	9.9 keV/μm	0.1	1.63	0.06	1.66	0.15
HUVEC	9.9 keV/μm	2	1.57	0.18	1.82	0.25
H460	9.9 keV/μm	0	1.39	0.04	1.39	0.30
H460	9.9 keV/μm	0.1	1.45	0.04	1.59	0.14
H460	9.9 keV/μm	1	1.89	0.35	1.90	0.35
H1299	9.9 keV/μm	0	1.30	0.05	1.56	0.37
H1299	9.9 keV/μm	0.1	1.37	0.06	0.91	0.07
H1299	9.9 keV/μm	1	1.24	0.11	1.25	0.22
PANC1	9.9 keV/μm	0	1.31	0.03	1.42	0.32
PANC1	9.9 keV/μm	0.1	1.44	0.04	1.44	0.15
PANC1	9.9 keV/μm	2	1.38	0.05	1.37	0.15
PANC10.05	9.9 keV/μm	0	1.42	0.06	1.36	0.42
PANC10.05	9.9 keV/μm	0.1	1.27	0.06	1.06	0.17
PANC10.05	9.9 keV/μm	2	1.38	0.10	1.66	0.31
MDA-MB-231	9.9 keV/μm	0	1.64	0.06	1.82	0.43
MDA-MB-231	9.9 keV/μm	0.1	1.70	0.09	2.01	0.23
MDA-MB-231	9.9 keV/μm	2	1.50	0.19	1.58	0.24
4T1	3.85 keV/μm	0	1.11	0.02	0.98	0.08
4T1	3.85 keV/μm	0.5	1.54	0.16	1.00	0.30
4T1	9.9 kevVμm	0	1.55	0.07	1.55	0.37
4T1	9.9 keV/μm	0.1	0.81	0.03	1.52	0.14
4T1	9.9 keV/μm	0.5	1.45	0.08	1.18	0.22

Supplementary Table 4. Summary of key resources.

Resource	Vendor	Catalogue Number
16% Paraformaldehyde Aqueous Solution	Electron Microscopy Sciences	15710
Goat serum	Abcam	ab7481
Tween-20	ThermoFisher	85113
Fish gelatin	MilliporeSigma	G7041
Fluoromount-G	ThermoFisher	00-4958-02
Triton-X	ThermoFisher	85111
53BP1 Antibody (polyclonal)	NOVUS biologicals	NB100-304
Anti-phospho-Histone H2A.X (Ser139) Antibody, clone JBW301	Millipore Sigma	JBW301
AZD6738	Selleckchem	S7693
BD Cytofix Fixation Buffer	BD Biosciences	554655
CD11b – Alexa Fluor 700	BioLegend	101222
CD11c – Brilliant Violet 510	BioLegend	117337
CD163 – PE	BioLegend	155308
CD4 – Brilliant Violet 510	BioLegend	100449
CD45 – APC/Fire750	BioLegend	103154
CD45 – Pacific Blue	BioLegend	103126
CD8a – PE/Cy7	BioLegend	100722
Cell strainer (70 μm)	Falcon	352350
DMEM	cytiva	SH30243
DMSO	EMD Millipore	317275
DNase	Sigma	D5025
Endothelial Cell Media	ScienCell	1001
F4/80 - PE/Dazzle 594	BioLegend	123146
FBS	Sigma	F0926
FxCycle™ PI/RNase Staining Solution	Invitrogen	F10797
HI-FBS	HyClone	SH30910
IFNγ – APC	BioLegend	505810
Ionomycin	Sigma	I9657
Liberase	Roche	05401127001
Lymphocyte Separation Medium	Corning	25-072-CV
PD1 – PE/Dazzle 594	BioLegend	135228
Perm buffer	Invitrogen	00-8333-56
Phorbol 12-myristate 13-acetate (PMA)	Sigma	P1585
Phospho-Histone H3 (Ser10) (D2C8) XP® Rabbit mAb (Alexa Fluor® 488 Conjugate)	Cell Signaling Technology	3465
Polyethylene-Glycol-300	Spectrum Chemical	P0108
Protein Transport Inhibitor (Golgistop)	BD Biosciences	51-2092KZ
Rad51 antibody [14B4]	Gentex	GTX70230
RPMI-1640	Corning	10-040-CV
TruStain FcX™ PLUS (anti-mouse CD16/32)	Biolegend	156604

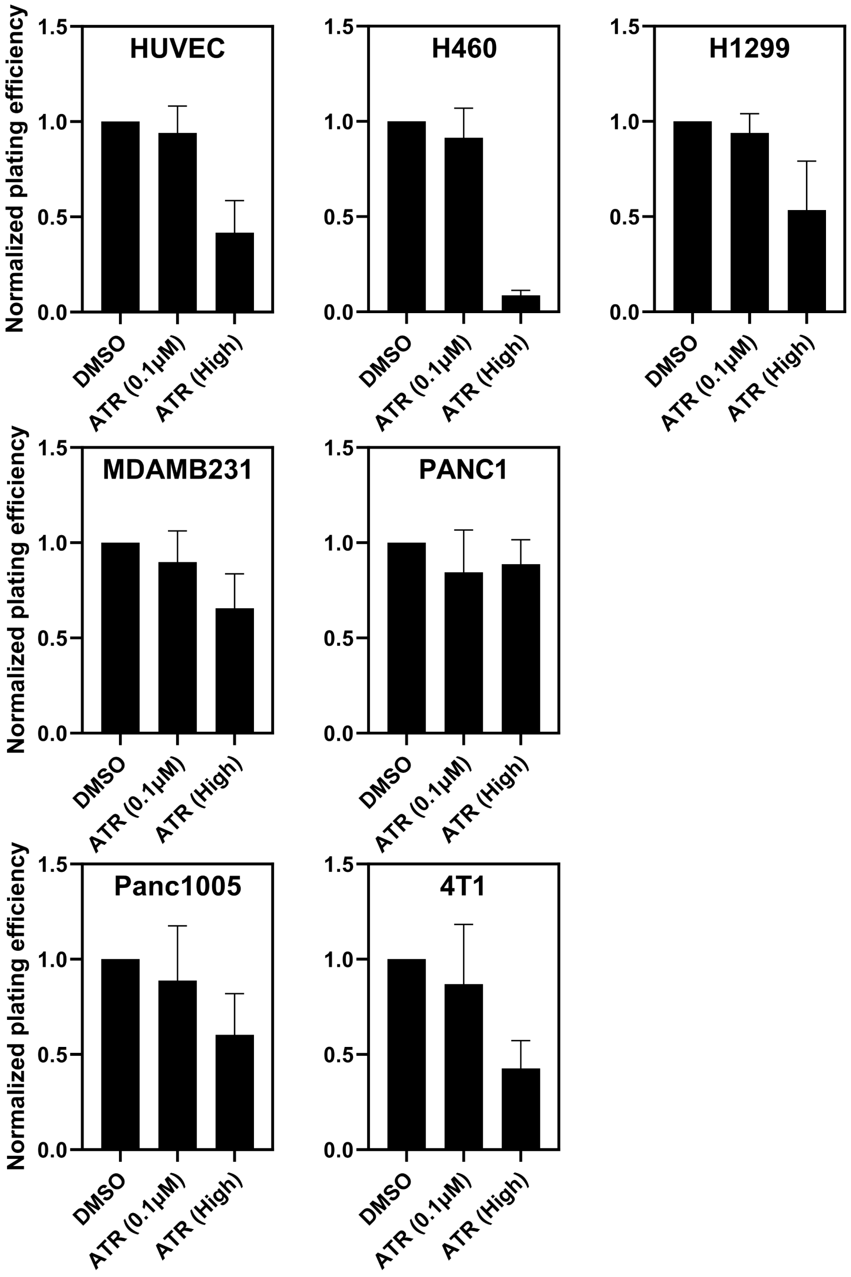

Supplementary Figure 1: Plating efficiency for each drug condition normalized to its respective DMSO condition.

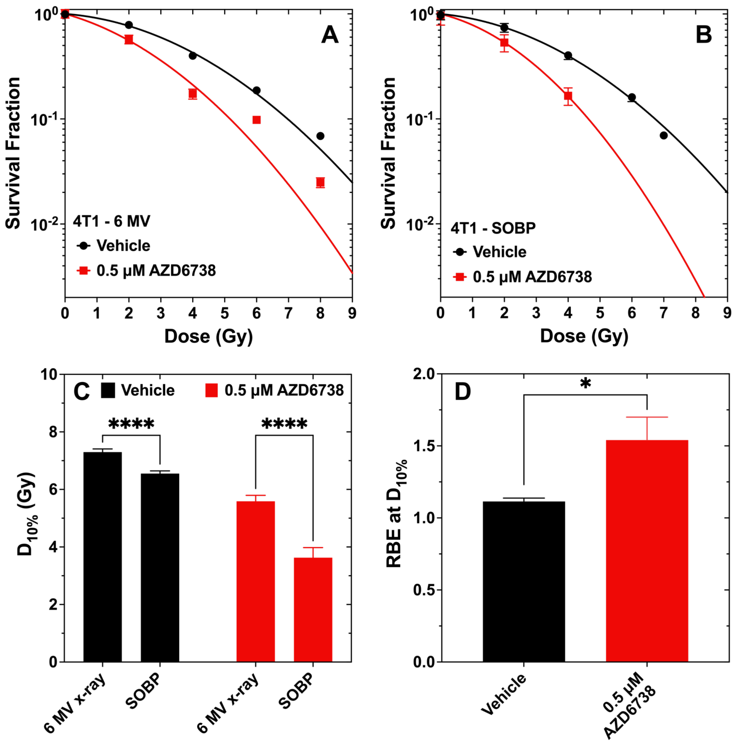

Supplementary Figure 2**.** Surviving fraction, $D_{10\%}$, and RBE. (A-B) 4T1 cell survival after exposure to photons or spread-out Bragg peak (SOBP) protons (3.85 keV/μm) (Supplementary Figure 6) with vehicle or 0.5 μM AZD6738. (C) $D_{10\%}$ for 4T1 cells exposed to photons or protons with or without AZD6738. (D) Relative biological effectiveness (RBE) at $D_{10\%}$ of vehicle and AZD6738 for SOBP protons. Error bars represent the SD. Statistical significance was assessed with two-way ANOVA with Tukey's multiple comparisons test (C) or unpaired t-test (D). ns: non-significant, *: P<0.05, **: P<0.01, ***: P<0.001, ****: P<0.0001.

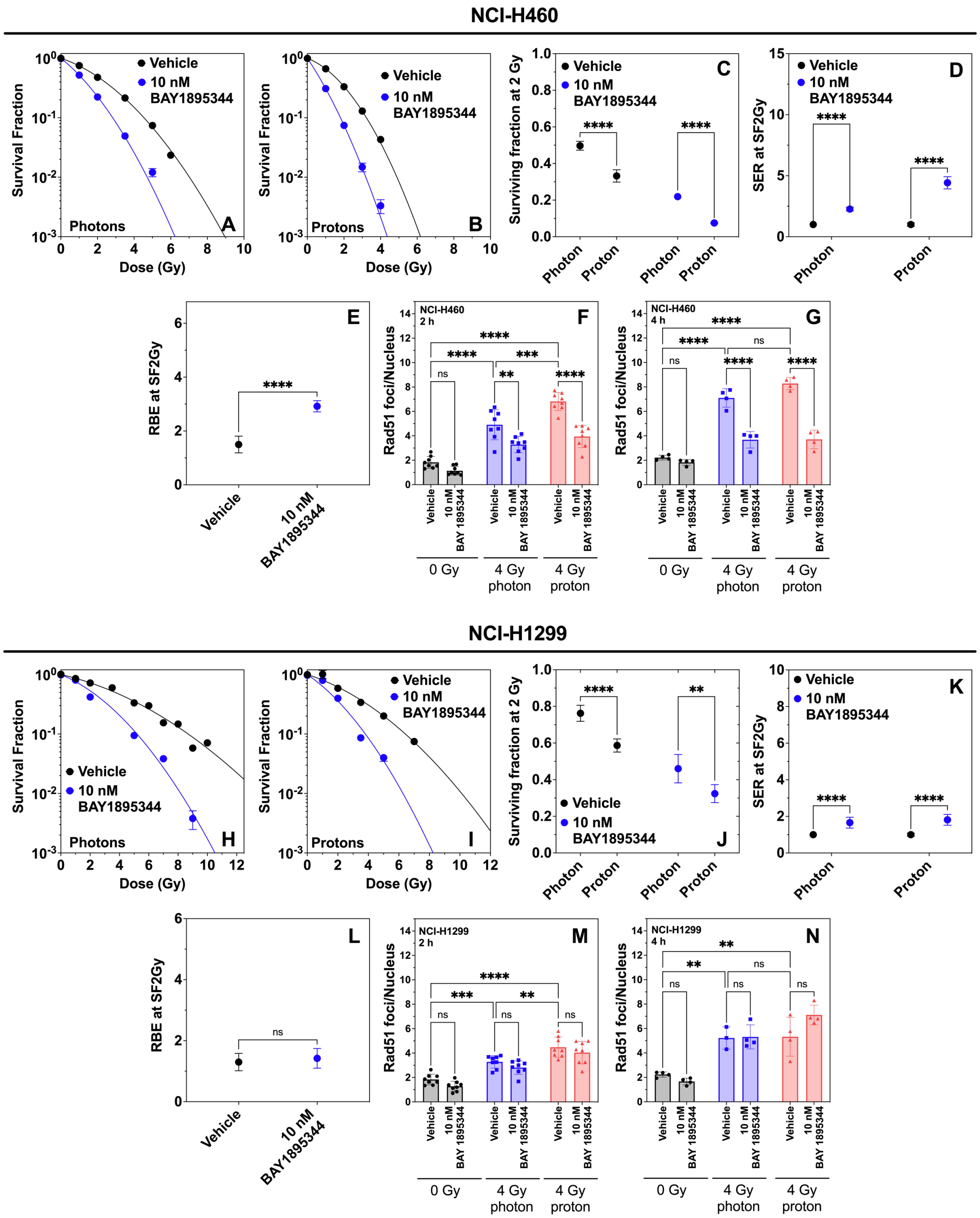

Supplementary Figure 3: Clonogenic survival curves of NCI-H460 and NCI-H1299 treated with photons and protons (A-D) combined with the ATR inhibitor BAY1895344. Surviving fraction was significantly reduced with BAY1895344 in both cell lines (E-F). Sensitization was significantly increased with BAY1895344. RBE was significantly increased for NCI-H460 (I) but not NCI-H1299 (J). RAD51 foci were significantly reduced with BAY1895344 in NCI-H460 (K-L) but not in NCI-H1299 (M-N). Error bars represent the SD. Statistical significance was assessed with two-way ANOVA with Tukey's multiple comparisons test (C) or unpaired t-test (D). ns: non-significant, *: P<0.05, **: P<0.01, ***: P<0.001, ****: P<0.0001.

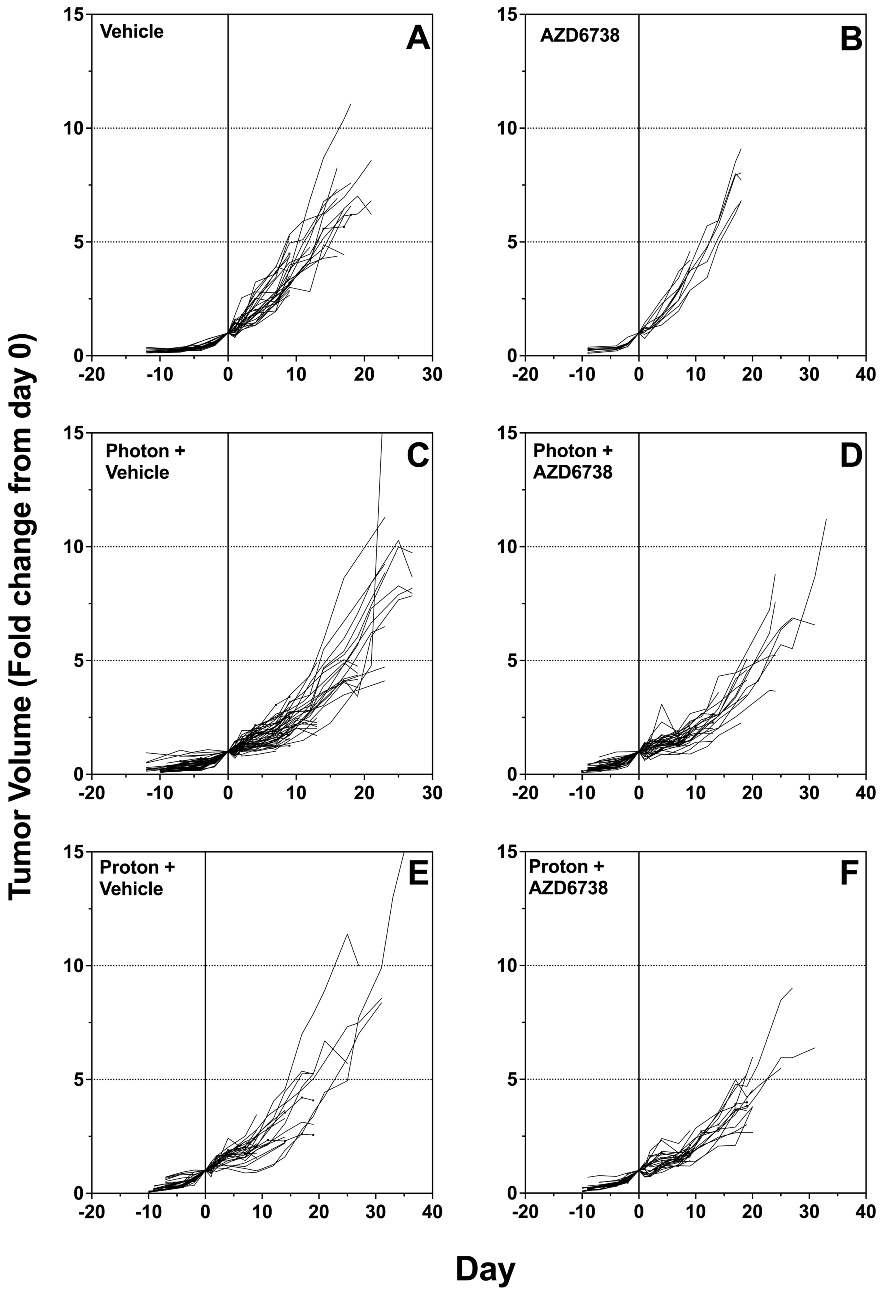

Supplementary Figure 4**.** Tumor growth delay in individual mice.

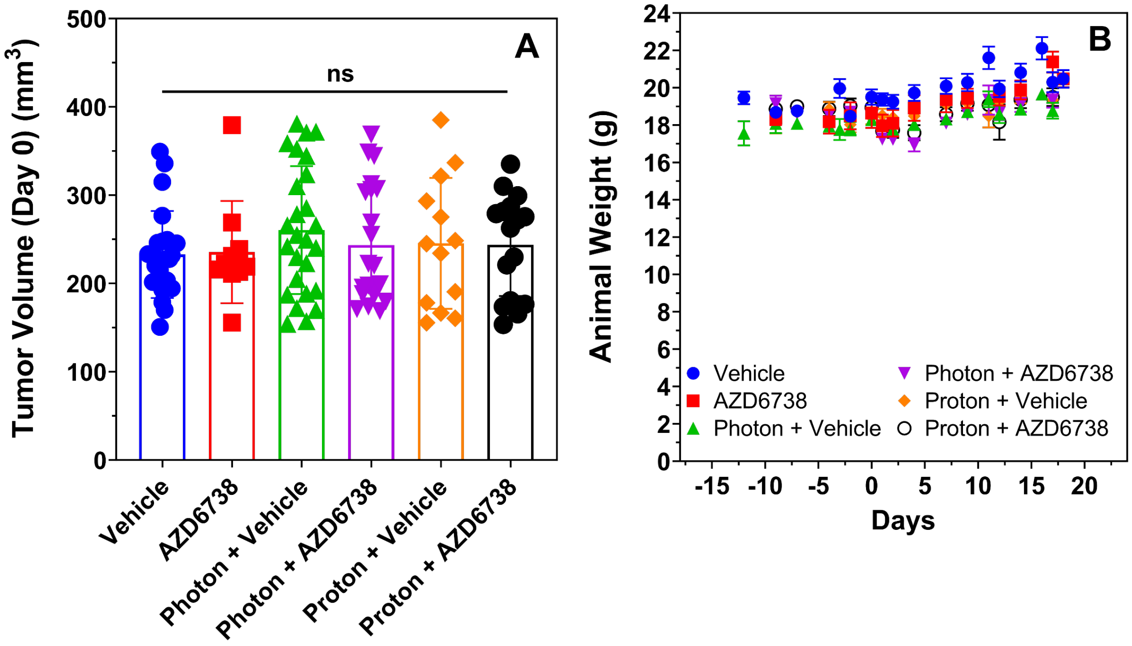

Supplementary Figure 5. Starting tumor volumes at day 0 and mean mouse body weights over time. Error bars represent the SD (A) or SEM (B). Statistical significance was assessed with two-way ANOVA with Tukey's multiple comparisons test. ns: non-significant, *: P<0.05, **: P<0.01, ***: P<0.001, ****: P<0.0001.

Supplementary Method 1. Irradiation conditions

Photon irradiations were done at the Proton Therapy Center in parallel with the proton irradiations so that cells were subjected to the same conditions except for radiation type. Photon irradiations were done with 6-MV x-rays (Truebeam, Varian Medical System), with a field size of 30 cm× 30 cm and gantry at 180º (beam from bottom to top). The beam crossed the couch, water equivalent blocks, and bottom of plates to a total water-equivalent thickness of 10 cm. Plastic blocks (>10 cm) were stacked upstream the plates to provide backscatter.

Proton irradiations were done with a double scattering nozzle with field size 18 cm × 18 cm (medium snout fully retracted), range (r_{90}) = 4.3 cm, E = 100 MeV (nominal energy), and gantry at 180º (beam from bottom to top). The beam crossed the couch, water equivalent blocks, and bottom of plates to a total WET of 4.42 cm. For spread-out Bragg peak irradiations, range (r_{90}) = 16.5 cm (with the use of range shifters), E = 200 MeV (nominal energy), SOBP = 4 cm, the beam crossed the couch, water equivalent blocks, and bottom of plates to a total WET of 15.6 cm.

For animal irradiations, mice were immobilized with custom-built restrainers that isolate the tumor-bearing hind leg. The restrainers are then placed in a jig that allows positioning of tumors of up to six mice in a water tank (31.2 cm length with 0.6-cm Lucite walls) so that clinical radiation beams could be delivered at a fixed depth with high precision and throughput.

Photon irradiations were done at the Proton Therapy Center in parallel with the proton irradiations so that animals were subjected to same conditions except for radiation type. Photon irradiations were done with 6-MV x-rays (Truebeam), field size 30 cm × 30 cm, and gantry at 0º and 270º. Parallel opposed beams were used to create a homogenous dose distribution. Tumors were positioned at a depth of 15.6 cm.

Proton irradiations were done with a double scattering nozzle with a field size of 25 × 25 cm^2 (large snout fully retracted), range (r_{90}) = 16.5 cm, E = 200 MeV (nominal energy), SOBP = 4 cm, gantry at 0º and 270º. Parallel opposed beams were used to create homogenous dose and LET distributions. The water tank and setup were the same as those used for photon irradiations.

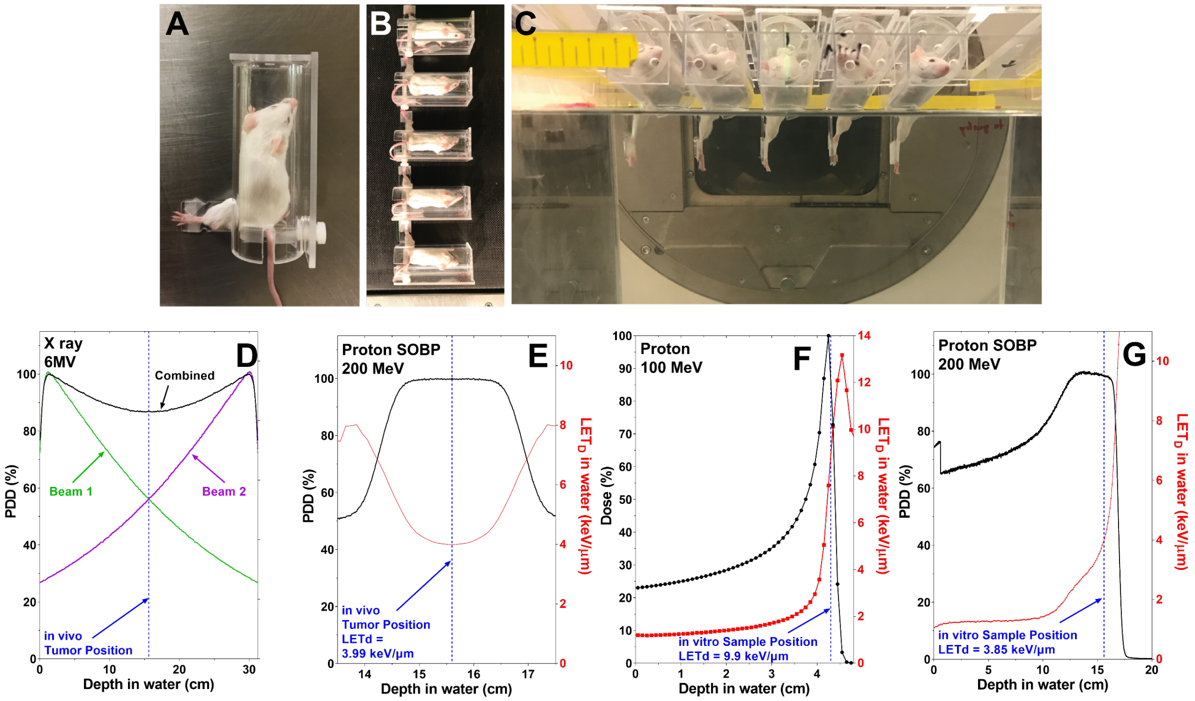

Supplementary Figure 6**.** (A) Restrainer that allows immobilization of the left leg of a mouse. (B-C) Jig that allows simultaneous irradiation of up to six mice with their tumors placed inside a water tank. A half-beam block is used to spare most of the mouse's body and internal organs from radiation. This setup allows each individual tumor to be precisely aligned to the radiation field. (D-E) We used this setup for 6-MV X-ray and proton irradiations with opposite lateral beams. The percentage depth dose and LET depth distributions in D-E are measured beam data for 6-MV photons and Monte Carlo simulated for protons. (F) In vitro proton irradiations were done with a 100-MeV (nominal energy) pristine beam with LET_D in water of 9.9 keV/µm. (G) We irradiated 4T1 cells in vitro and treated them with vehicle or AZD6738 to confirm radiosensitization in the LET range observed for our in vivo experiments; data are from Monte Carlo simulations. Samples were placed at the same depth as the tumors in our in vitro experiments.

Supplementary Method 2: Tumor-infiltrating lymphocyte extraction and analysis

Mice were euthanized in accordance with IACUC protocols at predefined times. The tumors were excised and kept in RPMI-1640 base medium until all samples were ready for processing. The base medium was removed, and the bulk tumor samples were manually separated into fine pieces in digestion buffer (RPMI-1640, 250 µg/ml Liberase, 20 µg/ml DNase I), and then kept at 37ºC with agitation for 30 minutes. The reaction was stopped with 1 mL of heat-inactivated fetal bovine serum before the samples were run through a 70 µm cell strainer. The filtered cell solution was centrifuged at 400 × *g* for 5 minutes. The cell pellet was collected and spun and washed with FACS buffer (PBS + 2% HI-FBS) by centrifugation at 400 × *g* for 5 minutes. The cell pellet was collected in PBS and immune cells were enriched by centrifugation with lymphocyte separation media for 30 minutes at room temperature at a speed of 400 × *g* (minimum acceleration and deceleration). The immune cells were collected and where incubated in RPMI-1640 (10% HI-FBS) with 0.5 µg/mL ionomycin, 50 ng/mL phorbol 12-myristate 13-acetate and protein transport inhibitor (Golgistop) for 4 hours at 37ºC. Cells were then washed and blocked with 0.5 µg TruStain FcX Plus for 20 minutes on ice. Antibodies were combined into a cocktail and added directly to the samples, which were then incubated for 20 minutes on ice in the dark. The cells were then washed twice with FACS buffer and centrifuged at 400 × *g* for 5 minutes. The cells were then fixed with BD Cytofix for 12 minutes on ice in the dark and stored overnight at 4ºC. The following day, cells were incubated with permeabilization buffer for 45 minutes at room temperature in the dark and then stained with antibodies for intracellular markers for 45 minutes in the dark at room temperature. Cells were washed twice in FACS buffer by centrifugation at 400 × *g* at room temperature and analyzed by flow cytometry at the MD Anderson Flow Cytometry and Cellular Imaging Core facility.

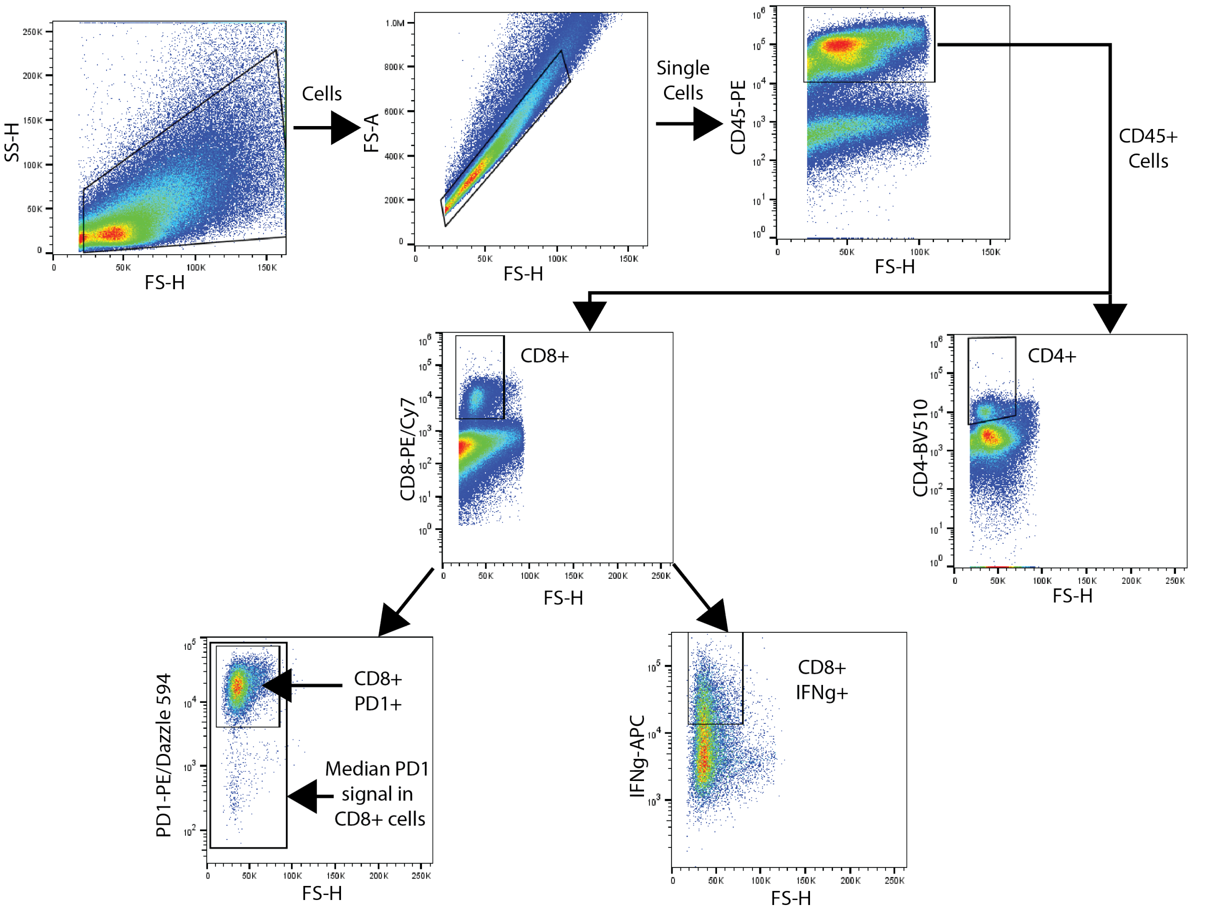

Supplementary Figure 7. Tumor-infiltrating lymphocyte gating strategy. Cells were initially gated for single cells and CD45^{+} immune cells before being gated for individual markers.

Supplementary Method 3: Method to calculate mean values of parameters of the clonogenic cell survival curves

For an individual clonogenic assay (replicates performed on the same day) we determined the plating efficiency first by computing the survival quotient for each dose, $SQ_D = N_{colonies,D} / N_{seeded,D}$. Then the SQ_D values were plotted versus dose and fit via variance weighted non-linear least squares minimization to an unnormalized version of the linear quadratic model: SQ = PE exp(-αD – βD2), where PE is the plating efficiency and α and β are free parameters. The plating efficiency determined from each experiment's fit was then used to calculate the survival fraction for each dose, $SF_D = SQ_D / PE$.

To combine data from multiple, biologically independent experiments, performed across time, we took the collection of all $SF_{D,combined}$ values measured across independent experiments, j and fit them simultaneously to the linear quadratic model:

$$SF_{combined,j}\ (D) = e^{-\alpha \cdot D - \beta \cdot D^2}$$

This ensures that the uncertainties reported from the final fit parameters incorporate both the inter- and intra-experimental variability present in the data while also properly accounting for the statistical power of the data set as a whole. In this work, to obtain the survival parameters for a given cell line's response to a particular condition, we fit the collection of all $SF_{D,combined,j}$ measured to the linear quadratic model via variance-weighted least squares minimization in Graph Pad Prism 10 (Graph Pad, San Diego, CA). This weighting ensures that the different confidences we have in the measured responses from different experiments are accounted for in the fit, while also ensuring, that our final fits are not insensitive to the data at lower survival levels. The uncertainties in the fit parameters provided, as well as their covariance, were obtained using Graph Pad Prism 10, and correspond to the elements of the covariance matrix associated with the fit. Survival metrics were calculated using the linear quadratic model for surviving fraction at 2 Gy or by rearranging the linear quadratic expression to:

$$D_{endpoint} = \frac{-\alpha \pm \sqrt{\alpha^2 - 4\beta \cdot \ln(SF_{endpoint})}}{2\beta}$$

for $D_{50\%}$ and $D_{10\%}$. This approach generates a single value for each survival curve metric, as opposed to taking the average of multiple values calculated from individual curves. Uncertainty for a given endpoint was calculated by using error propagation formula.

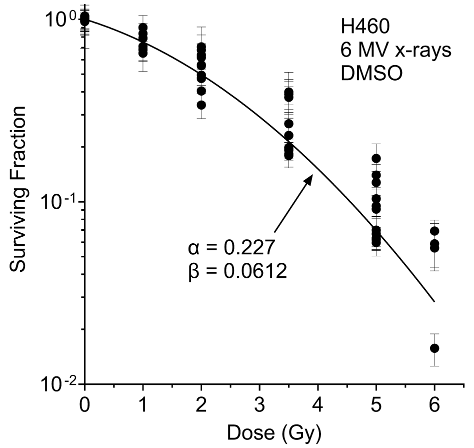

Supplementary Figure 8: To determine survival metrics including SF2Gy, $D_{10\%}$ and $D_{50\%}$, all replicates were fit to the linear quadratic model. Alpha and beta were determined from this global fit and used to generate the representative metric for a given condition.
